# TRIM31 promotes Atg5/Atg7-independent autophagy in intestinal cells

**DOI:** 10.1038/ncomms11726

**Published:** 2016-05-24

**Authors:** Eun A. Ra, Taeyun A. Lee, Seung Won Kim, Areum Park, Hyun jin Choi, Insook Jang, Sujin Kang, Jae Hee Cheon, Jin Won Cho, Ji Eun Lee, Sungwook Lee, Boyoun Park

**Affiliations:** 1 Department of Systems Biology, College of Life Science and Biotechnology, Yonsei University, Seoul 03722, South Korea; 2 Department of Internal Medicine and Institute of Gastroenterology, Yonsei University College of Medicine, Seoul 03722, South Korea; 3 Severance Biomedical Science Institute, Yonsei University College of Medicine, Seoul 03722, South Korea; 4 Department of Integrated OMICS for Biomedical Science, Yonsei University, Seoul 03722, South Korea; 5 Department of Health Science and Technology, Samsung Advanced Institute for Health Sciences and Technology, Sungkyunkwan University, Seoul 06351, South Korea; 6 Samsung Genome Institute (SGI), Samsung Medical Center, Seoul 06351, South Korea

## Abstract

Autophagy is responsible for the bulk degradation of cytosolic constituents and plays an essential role in the intestinal epithelium by controlling beneficial host–bacterial relationships. Atg5 and Atg7 are thought to be critical for autophagy. However, *Atg5*- or *Atg7*-deficient cells still form autophagosomes and autolysosomes, and are capable of removing proteins or bacteria. Here, we report that human TRIM31 (tripartite motif), an intestine-specific protein localized in mitochondria, is essential for promoting lipopolysaccharide-induced Atg5/Atg7-independent autophagy. TRIM31 directly interacts with phosphatidylethanolamine in a palmitoylation-dependent manner, leading to induction of autolysosome formation. Depletion of endogenous TRIM31 significantly increases the number of intestinal epithelial cells containing invasive bacteria. Crohn's disease patients display TRIM31 downregulation. Human cytomegalovirus-infected intestinal cells show a decrease in TRIM31 expression as well as a significant increase in bacterial load, reversible by the introduction of wild-type TRIM31. We provide insight into an alternative autophagy pathway that protects against intestinal pathogenic bacterial infection.

## Abstract (web summary)

While non-canonical, Atg5/Atg7-independent autophagy has been reported to occur, molecular details of alternative autophagy pathways remain unknown. Here, the authors report that the protein TRIM31 mediates alternative autophagy in intestinal cells, which protects against pathogenic bacteria.

## Graphical abstract

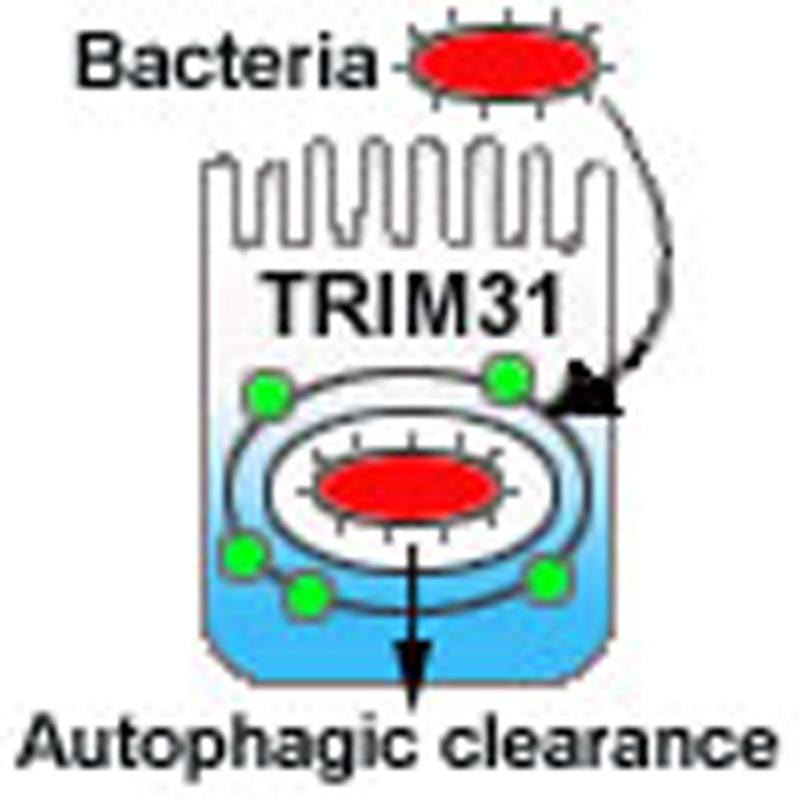

The intestinal epithelium interfaces with a dense microbial community and is an essential site for eliminating invasive bacteria and limiting further dissemination^1^. However, several invasive intestinal pathogens, such as *Salmonella Typhimurium* or *Shigella flexneri*, can achieve penetration of epithelial cells and threaten host intestinal health^2^,^3^. This suggests that intestinal cell-intrinsic immune responses are required to block invading bacteria and prevent their spread to extra-intestinal sites.

A primary role of autophagy is to maintain cellular homoeostasis by degrading intracytoplasmic proteins and organelles through starvation and by recycling multiple sources^4^,^5^,^6^. More recently, autophagy has been established as a critical process for the recognition and elimination of intracellular pathogens, thus acting as an important defensive barrier against bacterial invasion^7^,^8^. A genome-wide association study revealed several autophagy regulatory proteins with essential functions in intestinal immune homoeostasis^7^. In particular, mutations in the crucial autophagy protein Atg16L1 are tightly correlated with increased risk of Crohn's disease in humans^8^,^9^,^10^. However, a mutation in Atg16L1 still exhibits autophagy in mice^11^. In addition, it is well known that Atg5 and Atg7 are critical for autophagy^12^,^13^; however, equivalent numbers of LC3-positive and -negative autophagosomes were found in *Atg5*- or *Atg7*-deficient cells^14^. Furthermore, protein degradation or mycobacteria sequestration can occur through alternative Atg5- or Atg7-independent autophagy pathways^14^,^15^,^16^, indicating that canonical and non-canonical autophagy occur simultaneously. Particularly, intestinal tissue, which interfaces with a diversity of commensal bacteria, may actively mediate both conventional and alternative pathways to maintain intestinal immune homoeostasis.

The tripartite motif (TRIM) family of proteins, which contribute to a broad range of biological events, consist of a RING domain, one or two B-boxes and a coiled-coil domain^17^. Additionally, most TRIMs possess a variable C-terminal region, which may play a role in substrate binding. Although studies have shown that several TRIMs affect autophagy^18^,^19^,^20^, the functional role of the intestine-specific TRIM31 in autophagy has yet to be fully elucidated. Here, we report that TRIM31 plays a critical role in the formation of autolysosomes in *Atg5*^−/−^or *Atg7*^−/−^ cells, thus promoting elimination of invading bacteria through an Atg5- or Atg7-independent alternative pathway in the intestinal cells.

## Results

### TRIM31 is downregulated in Crohn's disease

**Figure 1:**
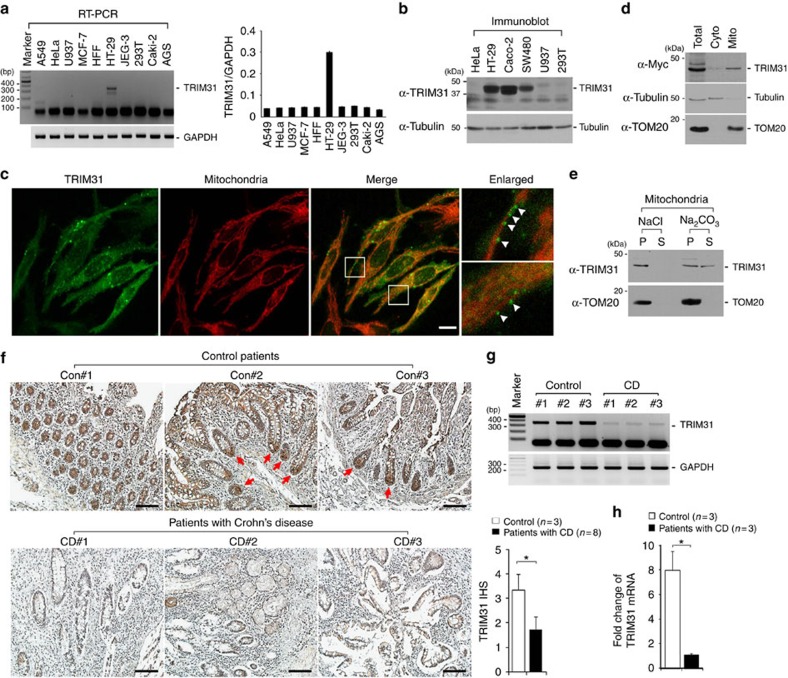
Human TRIM31 is enriched in the mitochondria and downregulated in Crohn's disease. (**a**,**b**) TRIM31 is highly expressed in colonic and intestinal epithelial cell lines. The expression of TRIM31 was analysed by RT-PCR (**a**) or immunoblot analysis (**b**) in various human cell lines: SW480 (colon), HT-29 (colon), and Caco-2 (intestine), A549 (lung), HeLa (cervix), U937 (lymphocyte), MCF-7 (breast), HFF (fibroblast), JEG-3 (placenta), 293T (embryonic kidney), Caki-2 (kidney) and AGS (stomach). Graph in **a**, quantification of TRIM31 mRNA levels normalized against GAPDH (mean±s.d.). (**c**) TRIM31 is enriched in the mitochondria. Immunofluorescence assay (IFA) of HeLa cells transfected with TRIM31-Myc. Cells were immunostained with anti-Myc antibody (green) and anti-TOM20 antibody (red), followed by AlexaFluor 488- or AlexaFluor 568-conjugated antibodies. TOM20 serves as mitochondrial marker. Scale bar, 5 μm. (**d**) Mitochondrial localization of TRIM31-Myc. Cytosolic and mitochondrial fractions were isolated from TRIM31-Myc-expressing HeLa cells, and then subjected to immunoblot analysis using anti-Myc antibody. Anti-TOM20 and anti-Tubulin antibodies were used to designate the mitochondrial and cytoplasmic compartments, respectively. (**e**) TRIM31 is associated with the mitochondrial membrane. Isolated mitochondria from TRIM31-Myc-expressing HeLa cells were treated with 0.1 M NaCl or 0.1 M Na_2_CO_3_ (pH 11.5) for 20 min on ice. After centrifugation, the supernatant (S) and pellet (P) were analysed by immunoblotting using anti-TRIM31 and anti-TOM20 antibodies. (**f**) Representative immunohistochemical analysis of human TRIM31 on tripled Crohn's disease (CD #1, #2, #3) and adjacent normal tissues from colon cancer (Con#1, #2, #3). The ileal mucosa biopsies of the distal small intestine from normal or Crohn's disease patients were stained with anti-TRIM31 antibody. Scale bars, 100 μm. Right graph, Immunohistochemical score (IHS) of TRIM31 in normal (*n*=3) or Crohn's disease patients (*n*=8). Detailed patient information is presented in Supplementary Table 2. IHS was equal to the quantity scores multiplied by the staining intensity scores. *P<0.01 (Student's *t*-test). (**g**) TRIM31 mRNA levels were determined by RT-PCR on tripled Crohn's disease (CD #1, #2, #3) and adjacent normal tissues from colon cancer (Con#1, #2, #3). (**h**) TRIM31 mRNA levels were normalized against GAPDH in the graph. *P<0.01 (Student's *t*-test). All data are representative of at least three independent experiments (mean±s.d. in **f**,**h**).

A recent study has shown that TRIM31 expression in human tissues is limited to digestive tissues such as the colon and small intestine among normal tissues^21^. Consistent with a previous report, our data confirmed this tissue-specific expression of TRIM31 in human colonic and intestinal epithelial cells among various human cell lines (Fig. 1a,b). Because TRIM family proteins are distributed in a variety of cellular compartments involved in diverse biological processes, we first sought to determine the subcellular localization of TRIM31 to understand its function. We tested various subcellular markers for the endoplasmic reticulum, Golgi apparatus, nucleus, cytoskeleton, lysosomes and mitochondria in HeLa and intestinal epithelial cells. Most of the surveyed organelles exhibited little or no TRIM31 expression; however, TRIM31 was found associated with the mitochondria and lysosomes as punctate structures (Fig. 1c; Supplementary Fig. 1a,b). Interestingly, high-resolution imaging showed that the TRIM31-containing structures were in close proximity with mitochondria and dangled from mitochondrial tubules (Fig. 1c, arrowheads). To confirm these results, we performed subcellular fractionation for mitochondria and the cytosol. TRIM31 was found enriched in the mitochondrial fraction with a very small percentage of the protein found in the cytoplasmic fraction (Fig. 1d). We next examined whether mitochondria-targeted TRIM31 is incorporated in or attached to mitochondria. To test this, we treated mitochondria at alkaline pH, conditions that lead to the extraction of soluble and peripheral membrane proteins, whereas integral membrane proteins remain incorporated^22^,^23^. TRIM31 was highly enriched in the soluble fraction after alkalization, which is different from membrane-integrated TOM20, suggesting that TRIM31 is attached to the mitochondria (Fig. 1e).

To explore the functional roles of TRIM31 in intestine, we next examined TRIM31 expression in samples from patients with Crohn's disease or controls. Crohn's disease, a type of inflammatory bowel disease (IBD) in which inflammation is localized to the distal small intestine and variable regions in the colon^7^. Interestingly, TRIM31 was distributed throughout the ileal epithelium and predominantly enriched in the lower regions of the crypts, which contain Paneth cells (Fig. 1f, red arrows). Previous reports demonstrated that Crohn's disease leads to destruction of the intestinal crypt^7^,^24^,^25^, which we also observed in the crypt regions (Fig. 1f, bottom panel). Remarkably, TRIM31 expression decreased by ∼45% in Crohn's disease patients compared with normal ileum tissues (Fig. 1f, right graph). Additionally, in contrast to normal samples, the level of TRIM31 mRNA in Crohn's disease patients decreased by 85%, suggesting that TRIM31 is downregulated in Crohn's disease due to a defect in transcription (Fig. 1g,h). Taken together, these results suggest that TRIM31 expression is tightly controlled in a tissue-specific manner, and expression of TRIM31 may associate with the epithelial function of the intestine mucosa or intestinal immune homoeostasis.

### TRIM31 induces autophagy

**Figure 2:**
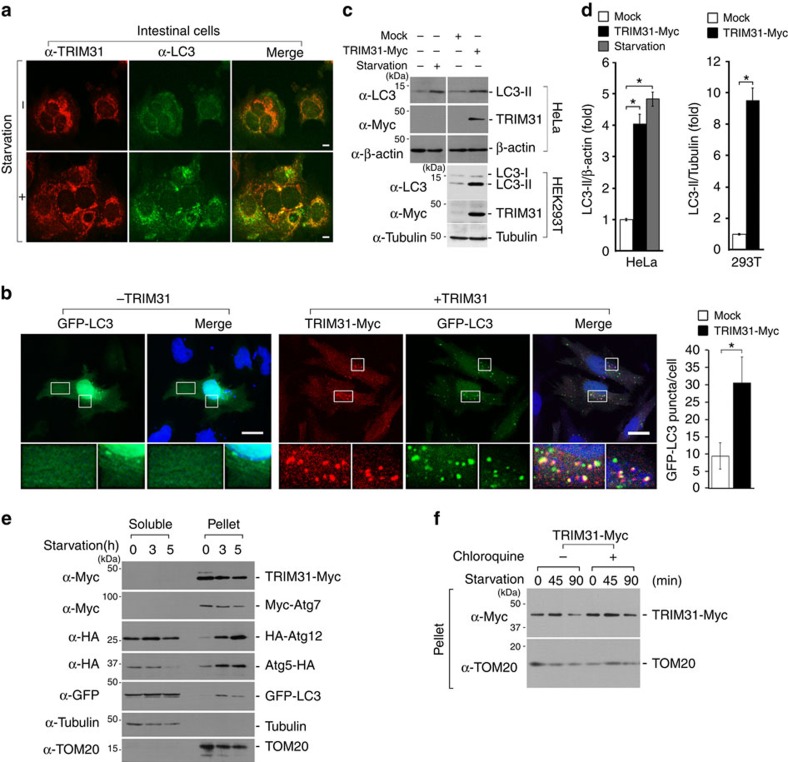
Human TRIM31 induces autophagy. (**a**) Starvation enhances colocalization of endogenous TRIM31 and LC3 in the intestinal epithelial cell line, Caco-2. Cells were incubated with complete media (-starvation) or serum-free DMEM for 6 h, immunostained with anti-TRIM31 and anti-LC3 antibodies, and then analysed by confocal microscopy. Scale bars, 5 μm. (**b**) TRIM31-Myc (red) colocalizes with GFP-LC3 (green) and accumulates LC3-positive puncta in HeLa cells. TRIM31-Myc was stained using anti-Myc antibody and analysed by IFA. Blue, DAPI. Scale bars, 5 μm. Right graph, quantification of LC3 puncta in HeLa cells expressing either Mock or TRIM31-Myc. *P<0.01 (Student's *t*-test). (**c**) Overexpressed TRIM31-Myc upregulates LC3-II in HeLa (upper panels) and HEK293T (bottom panels) cells. HeLa cells were starved in serum-free DMEM for 8 h and then subjected to immunoblot analysis with the indicated antibodies. (**d**) The LC3-II band intensities in **c** were quantified by scanning densitometry and normalized to the β-actin or tubulin band intensity. *P<0.01 (Student's *t*-test). (**e**) Starvation leads to the enrichment of autophagosomal components in the pellet fraction of HeLa cells. HeLa cells were transfected with plasmids expressing TRIM31-Myc, Myc-Atg7, HA-Atg12, Atg5-HA or EGFP-LC3 and then starved in serum-free DMEM for the indicated times. Cells were separated into soluble and pellet fractions by centrifugation, and then subjected to immunoblot analysis with the indicated antibodies. TOM20 and Tubulin were used to indicate the mitochondrial and cytoplasmic compartments, respectively. (**f**) TRIM31-Myc-expressing HeLa cells were starved in serum-free DMEM and then incubated with chloroquine. Cells were lysed with 1% NP-40 and separated into soluble and pellet fractions by centrifugation. Samples were subjected to SDS–PAGE and detected with primary antibodies as indicated. All data are representative of at least two independent experiments (mean±s.d. in **b**,**d**).

Recent studies suggest that mitochondria can participate autophagosome biogenesis by supplying the membrane source^26^. In addition, Paneth cells play a role in control of intestinal microbiota by secretion of antimicrobial peptides and by regulation of autophagy process^11^,^27^. TRIM31 was enriched in the mitochondria and its aberrant expression with very low intensity was shown in samples from Crohn's disease patients (Fig. 1f–h), leading us to focus our investigation to TRIM31 and its role in autophagy. We therefore examined whether the TRIM31-positive puncta overlap with expression of the late autophagosomal marker, LC3. Under normal conditions, LC3 is distributed between the cytoplasm and nucleus with very little detected on autophagic vesicles. Once induced by a starvation response, LC3 is recruited to autophagosomal membranes^28^,^29^. Unexpectedly, endogenous TRIM31-positive structures partially overlapped with LC3-positive autophagosomes under normal conditions (Fig. 2a, upper panel). We also found that starved intestinal cells displayed dramatic accumulation of TRIM31-expressing structures, which overlapped with LC3 staining (Fig. 2a, lower panel). Furthermore, HeLa cells overexpressing TRIM31 showed intense LC3-specific staining even under normal conditions (Fig. 2b). Cells treated with chloroquine, a lysosomal inhibitor that leads to an accumulation of autophagic vacuoles, produced an intense, clumped and vesicular pattern of TRIM31 staining relative to untreated cells, which exhibited relatively weak TRIM31 staining (Supplementary Fig. 2a). Additionally, because degradation of p62 has been used as a marker for the induction of autophagy^30^,^31^,^32^, we measured the level of endogenous p62 as a function of TRIM31-mediated autophagic activity. Consistent with previous results, TRIM31-expressing cells showed a significant increase in p62 degradation (Supplementary Fig. 2b). Thus, these findings indicate that TRIM31 may be directly involved in autophagy.

To clarify the involvement of TRIM31 on autophagy, we assessed the conversion of LC3-I to LC3-II in TRIM31-overexpressing cells. Notably, expression of Myc-tagged TRIM31 significantly induced an accumulation of LC3-II even in the absence of starvation (Fig. 2c,d), suggesting that human TRIM31 induces autophagy even under nutrient-replete conditions.

Because the conserved Atg5–Atg12–Atg16 complex binds directly to the pre-autophagic membranes, we examined whether autophagic membranes also contain TRIM31. We fractionated TRIM31- or autophagy-related proteins-expressing cells and these proteins were recovered in the pellet and soluble fractions. As reported previously^28^,^29^,^33^,^34^,^35^, the level of Atg7, Atg12, Atg5 and LC3 were highly enriched in the pellet fractions (Fig. 2e). In a similar way, this separation resulted in the exclusive partitioning of TRIM31 to the pellet fractions (Fig. 2e, first panel). In addition, these isolated pre-autophagic membranes also contained mitochondria derived, but not cytoplasm-derived markers, suggesting that the autophagic membranes originating from mitochondria contain TRIM31. In particular, the amount of LC3 and TRIM31 proteins gradually decreased in the pellet after 3 h of starvation, possibly due to autolysosomal degradation during autophagy process (Fig. 2e, first and fifth panels). To test this, we administered chloroquine to TRIM31-Myc-expressing cells and examined the level of TRIM31 expression. As expected, substantial TRIM31 accumulation was observed in the pellet of chloroquine-treated cells (Fig. 2f; Supplementary Fig. 2c), Taken together, these findings suggest that TRIM31 is involved in the autophagy process.

### TRIM31 promotes autophagy induced by LPS

**Figure 3:**
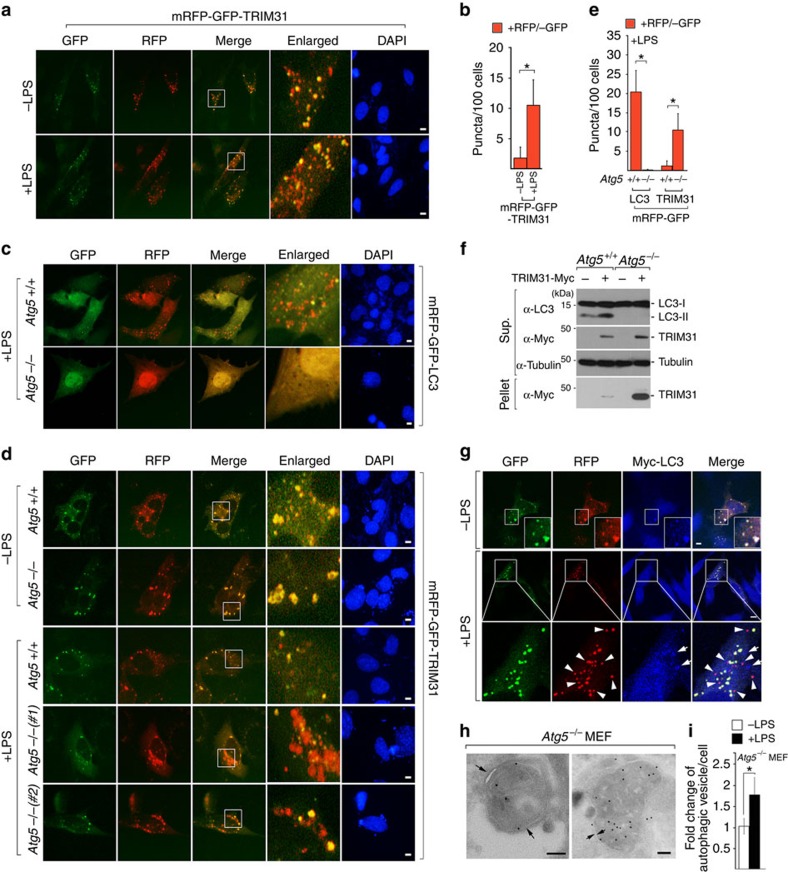
TRIM31 induces LPS-induced Atg5/7-independent alternative autolysosome formation. (**a**) HeLa cells expressing mRFP-GFP-TRIM31 were left untreated or treated with LPS (100 ng ml^−1^) for 4 h. Blue, DAPI. Scale bars, 5 μm. (**b**) Quantification of red puncta is shown in the graph. *P<0.01 (Student's *t*-test). (**c**,**d**) Wild-type or *Atg5*^−/−^ MEF cells were transfected with mRFP-GFP-LC3 or mRFP-GFP-TRIM31. After 24 h, cells were stimulated with LPS (100 ng ml^−1^) for 10 h and analysed by IFA. Blue, DAPI. Scale bars, 5 μm. (**e**) Quantification of red puncta is shown in the graph. *P<0.01 (Student's *t*-test). (**f**) Cell extracts from wild-type or *Atg5*^−/−^ cells expressing Mock or TRIM31-Myc were fractionated into pellets and supernatants by centrifugation. Detergent-insoluble pellets were resuspended in 1 × SDS loading buffer and loaded onto SDS–PAGE gels with the same amount of supernatant, and then analysed by immunoblot analysis with the indicated antibodies. Tubulin was used as a loading control. (**g**) HeLa cells expressing mRFP-GFP-TRIM31 and Myc-LC3 (blue) were left untreated or treated with LPS (100 ng ml^−1^) for 4 h. Scale bar, 5 μm. (**h**) RING deletion mutant of TRIM31 in *Atg5*^−/−^ MEFs is present on autophagosomal (left) and autolysosomal (right) structures. *Atg5*^−/−^ cells expressing TRIM31-ΔRING-Myc were stimulated with LPS (100 ng ml^−1^) for 3 h with chloroquine (5 μM). The localization of TRIM31-ΔRING-Myc was examined by immunogold electron microscopy using anti-Myc antibody. Arrows indicate double-membrane structure. Scale bar, 0.1 μm. (**i**) *Atg5*^−/−^ cells expressing TRIM31-ΔRING-Myc were left untreated or treated with LPS (100 ng ml^−1^) for 3 h. Quantification of autophagosomal vesicles is shown in the graph. *P<0.01 (Student's *t*-test). All data are representative of at least two independent experiments (mean±s.d. in **b**,**e** and **i**).

We then proceeded to clarify whether TRIM31-containing puncta indeed constitute an autophagic structure that subsequently fuses with lysosomes to form autolysosomes. To accomplish this, we used tandem green fluorescent protein (GFP) and red fluorescent protein (RFP)-tagged LC3 and TRIM31 fusion proteins. Whereas GFP is sensitive to acidic conditions, monomeric RFP is resistant to both acid and lysosomal proteases. These properties allow the red and green fluorescence of mRFP-GFP-LC3 to be sustainable and transient, respectively, thereby providing us with a valuable tool for monitoring the maturation process from autophagosome to autolysosome^36^,^37^. In particular, because lipopolysaccharide (LPS) derived from the cell wall of Gram-negative bacteria induces autophagy^38^,^39^, we assessed LPS-mediated autolysosomal formation to understand the functional role of human TRIM31 in intestinal epithelial autophagy. Yellow puncta, reflective of combined GFP and RFP fluorescence, represent autophagosomes, whereas red puncta represent autolysosomes whose acidic pH quenches GFP fluorescence. Under LPS-stimulated conditions, an increase in the red signal of LC3 was observed with the yellow signal (Supplementary Fig. 3a,b). Remarkably, TRIM31 RFP signals were significantly increased in LPS-stimulated cells, indicating that incorporation of TRIM31 into autolysosomes was increased in response to LPS (Fig. 3a,b).

We further examined whether the formation of TRIM31-positive autolysosomes shares the E3-like enzyme, Atg5–Atg12, similar to the conventional autophagy process. As previously reported in many studies, *Atg5*^−/−^ mouse embryonic fibroblasts (MEFs) failed to form autolysosomes, whereas LC3-containing autolysosomes developed in control cells (Fig. 3c). Surprisingly, *Atg5*^−/−^ cells demonstrated formation of numerous TRIM31-positive autophagosomes under normal conditions. Moreover, following LPS stimulation, a significant increase in red signal was observed with little decrease in the yellow signal (Fig. 3d,e). TRIM31 expression was also significantly increased in *Atg5*^−/−^ cells, especially in the pellet fractions, indicating that TRIM31-mediated autophagy may compensate for the loss of Atg5 in the conventional process by inducing TRIM31 expression (Fig. 3f). Furthermore, while TRIM31-associated red dots colocalized with LC3 in the absence of LPS, large numbers of TRIM31 RFP signal did not overlap with LC3 in LPS-stimulated cells (Fig. 3g, arrowheads). Taken together, these results suggest that TRIM31 can induce autolysosomes via an Atg5-independent alternative autophagic pathway.

To examine the localization of TRIM31 precisely, we carried out immune-electron microscopy (EM) using an anti-Myc antibody. Because most studies use an overexpression system to visualize the subcellular localization of autophagic components by EM, we generated *Atg5*^−/−^ cells, which stably expressed TRIM31-Myc. However, we failed to maintain a high enough level of TRIM31 expression in LPS-treated cells since we observed a rapid degradation of TRIM31 in LPS-exposed cells (Supplementary Fig. 4a). Although this degradation was attenuated by chloroquine, it was not sufficient for the optimal expression levels of TRIM31. Thus, we hypothesized that TRIM31 may degrade in a proteasome-dependent manner together with autolysosomes during LPS stimulation because TRIM31 belongs to the TRIM family, which contributes to degradation of target proteins through ubiquitination^21^. Indeed, TRIM31 degradation was inhibited by the proteasome inhibitor, MG132, indicating that TRIM31 may undergo ubiquitination itself, leading to proteasome-dependent degradation (Supplementary Fig. 4b). To test this hypothesis, we generated a TRIM31 deletion mutant lacking the RING domain (Supplementary Fig. 4c). Compared with wild-type TRIM31, the deletion mutant abolished the auto-ubiquitination activity that leads to prolonged TRIM31 expression (Supplementary Fig. 4d). In addition, this mutant did not affect TRIM31-positive autophagic formation or phosphatidylethanolamine (PE) interaction, as seen with wild-type TRIM31 (Supplementary Fig. 4e,f); this result enabled us to maintain a high level of TRIM31 expression in LPS-stimulated *Atg5*^−/−^ cells for observing its subcellular localization by EM. Notably, TRIM31 was clearly observed in typical double-membrane autophagic structures in LPS-stimulated *Atg5*^−/−^ cells with chloroquine, as TRIM31 RFP signals increased significantly in LPS-stimulated cells (Fig. 3h). Furthermore, we observed an approximately twofold increase in the total number of autophagic vesicles in LPS-stimulated *Atg5*^−/−^ cells expressing TRIM31 compared with untreated cells (Fig. 3i). Therefore, we conclude that TRIM31 promotes LPS-dependent autophagy in an Atg5-independent alternative process.

### TRIM31 interacts with PE independently of Atg7

**Figure 4:**
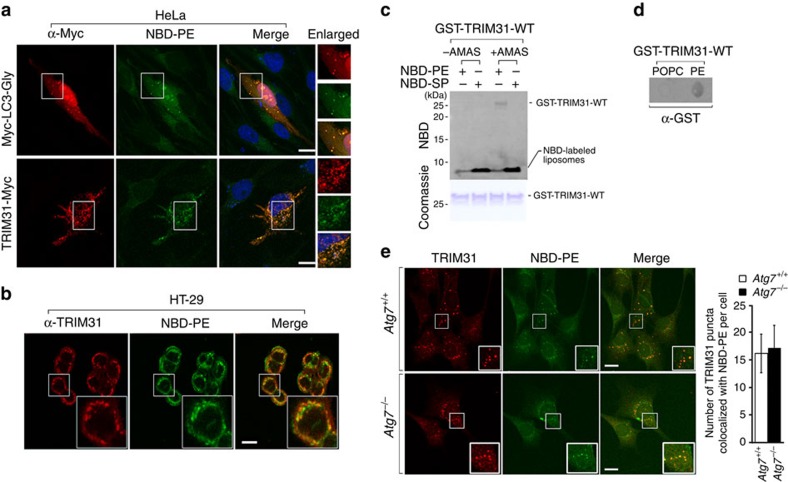
TRIM31 interacts with PE in an Atg7-independent manner. (**a**) Colocalization of NBD–PE with TRIM31-Myc in HeLa cells. Intracellular NBD–PE labelling using delivery of liposomes consisting 80% 18:1–16:0 PC and 20% 18:1–12:0 NBD–PE. Cells were exposed to liposomes, washed with PBS, and maintained in complete media for 2 h. The C-terminal glycine-exposed form of LC3, Myc-LC3-Gly, was used as a positive control. (**b**) Colocalization of NBD–PE with endogenous TRIM31 in HT-29 cells. Cells were exposed to NBD–PE liposomes and fixed. Cells were then immunostained with an anti-TRIM31 antibody. Scale bars, 5 μm. (**c**) Crosslinking experiment of purified GST-tagged TRIM31-wild-type B-Box (GST-TRIM31-WT) with liposomes consisting of 20% NBD–PE/80% POPC or 20% NBD-sphingosine/80% POPC. Liposomes were incubated with the cross-linker AMAS (0.5 mM), and then glycine was added to terminate the crosslinking reaction. GST-TRIM31-WT was added to the reaction buffer, and sample reactions were terminated with the addition of 5 × SDS loading buffer. The LAS image analyser was used to detect NBD fluorescence (upper) while Coomassie blue staining was used to visualize protein levels (bottom). (**d**) The ability of the GST-tagged TRIM31-wild-type B-Box (2 μg ml^−1^) to bind PE or POPC was analysed by a protein-lipid binding assay on nitrocellulose. Nitrocellulose membranes were spotted with 100 pmol of phospholipid. (**e**) TRIM31-Myc colocalizes with NBD–PE in puncta independent of Atg7. Wild-type or *Atg7*^−/−^ cells stably expressing TRIM31-Myc were labelled with liposomes containing 20% NBD–PE and 80% PC and chased in complete media for 1 h, followed by immunostaining with anti-Myc antibody. Scale bars, 5 μm. Quantification is shown in the right graph (mean±s.d.). All data are representative of at least three independent experiments.

Mitochondria are a primary source of membranes, where phosphatidylserine is converted to PE by the mitochondrial enzyme phosphatidylserine decarboxylase^26^. Additionally, since LC3 conjugation to PE is essential for membrane tethering and isolated membrane expansion during autophagosome formation^40^,^41^, we hypothesized that mitochondria-localized TRIM31 may directly interact with PE for autolysosome formation. To test this, we treated cells with 80% 18:1–16:0 PC (1-oleoyl-2-palmitoyl-*sn*-glycero-3-phosphocholine) and 20% 18:1–12:0 nitrobenzosadiazole (NBD)–PE (1-oleoyl-2-{12-[(7-nitro-2-1,3-benzoxadiazol-4-yl)amino]dodecanoyl}-*sn*-glycero-3-phosphoethanolamine), a fluorescent analogue of PE, and then assessed NBD–PE localization. The fluorescent signal of NBD–PE was observed in the endoplasmic reticulum membrane early after cell loading and subsequently within mitochondrial membranes (Supplementary Fig. 5a). In cells expressing LC3-Gly, in which the C-terminal glycine residue was exposed, NBD fluorescence clearly appeared in LC3-Gly-positive autophagosomal puncta, suggesting that NBD–PE can transfer from mitochondria to autophagosomes (Fig. 4a, upper panel). Interestingly, a strong signal in NBD–PE also overlapped with TRIM31 (Fig. 4a, lower panel). In addition, colonic epithelial cells clearly exhibited colocalization of TRIM31 with NBD–PE (Fig. 4b). To further determine how each domain contributes to TRIM31–PE colocalization, we generated truncated forms of Myc-tagged TRIM31 (Supplementary Fig. 5b). Both TRIM31ΔN and TRIM31ΔC still formed TRIM31-positive puncta that overlapped with NBD–PE in a similar manner as wild-type TRIM31. Surprisingly, TRIM31ΔBB was only distributed throughout the cytoplasm and therefore failed to form a punctate structure with NBD–PE (Supplementary Fig. 5c). These findings suggest that the B-box domain is required for the formation of TRIM31-positive puncta and colocalization with PE.

To analyse the direct interaction between TRIM31 and PE, we used a liposome-based cell-free system that enables chemical interaction of TRIM31 to PE lipids, thus bypassing the requirement for any conjugation machinery^42^. This system is based on a crosslinking reaction between substrates and the PE lipid. Notably, TRIM31 interacted with PE but not sphingosine as demonstrated by measuring the NBD fluorescence signal in the presence of crosslinker (AMAS) (Fig. 4c). To support these results, we performed a protein-lipid overlay assay, as described previously^43^,^44^. Indeed, TRIM31 bound strongly to PE but not 1-palmitoyl-2-oleoyl-sn-glycero-3-phosphocholine (POPC; Fig. 4d). Collectively, these results suggest that TRIM31 specifically interacts with PE.

Since studies have established that LC3 lipidation is mediated by an amide bond between the exposed C-terminal glycine and the amine group of PE^33^, we examined whether TRIM31 is covalently conjugated to PE. We found that TRIM31 possesses two glycines, which are highly conserved across species (Supplementary Fig. 6a). To test whether glycine residues on TRIM31 are involved in PE interaction, we constructed two truncation mutants, TRIM31-110G and TRIM31-163G, in which the glycine residues were made available for covalent coupling with PE (Supplementary Fig. 6b). Our examination revealed that these mutants were distributed throughout the nucleus or cytoplasm and failed to detect any punctate structures in a similar pattern as TRIM31ΔBB (Supplementary Fig. 6c), suggesting that the TRIM31–PE interaction is not mediated by glycine-dependent covalent binding.

We also determined whether the TRIM31–PE interaction is mediated by the E1-like enzyme, Atg7, which participates in the conventional autophagy process. In the case of LC3, binding to PE was observed in control cells, whereas this interaction was absent in *Atg7*^−/−^ cells, due to failure to form LC3-positive autophagosomes (Supplementary Fig. 7a). We have previously shown that *Atg5*^−/−^ cells form numerous TRIM31-mediated autophagosomes/autolysosomes (Fig. 3). Notably, the TRIM31–PE interaction was clearly observed in *Atg7*^−/−^ cells (Fig. 4e). We also examined TRIM31–PE binding in HT-29 cells lacking Atg7, Beclin1, or LC3. As expected, TRIM31 clearly bound to PE, even in the absence of canonical autophagy-related proteins (Supplementary Fig. 7b). Therefore, mitochondrial TRIM31 can directly interact with PE, thereby enabling autolysosome formation, independent of the conventional autophagy pathway.

### Palmitoylation is involved in autophagy mediated by TRIM31

**Figure 5:**
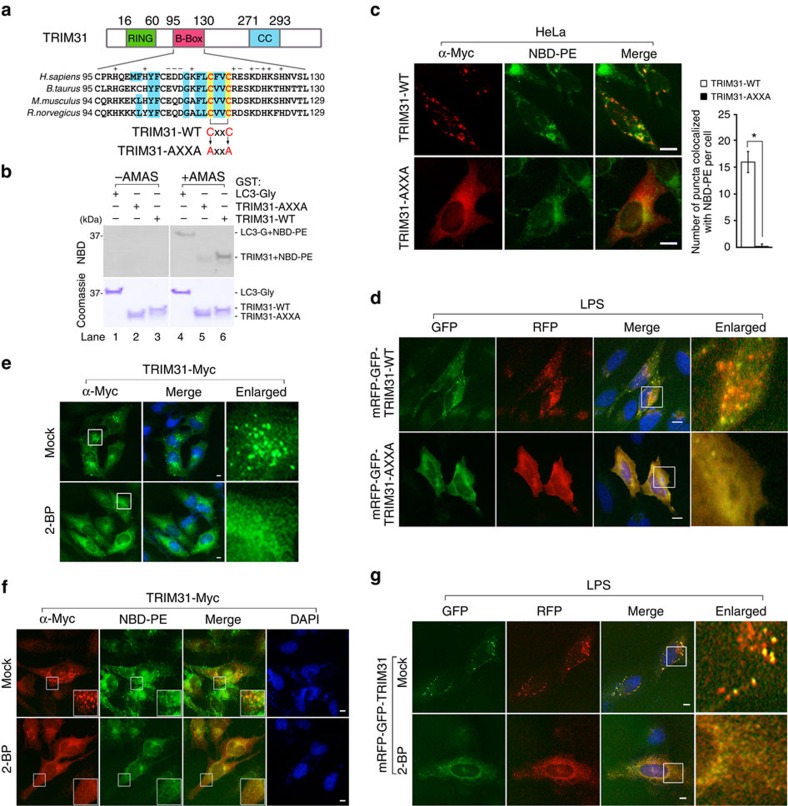
TRIM31 interacts with PE in a palmitoyl-dependent manner. (**a**) Multiple sequence alignment of the B-box domain containing a predicted palmitoylation site composed of the conserved CXXC motif. The wild-type TRIM31 CXXC motif (TRIM31-WT) was mutated to AXXA (TRIM31-AXXA). Blue box, conserved hydrophobic amino acids; yellow box, conserved cysteine residues. The RING, B-box and coiled-coil domains are depicted in green, pink and blue, respectively. (**b**) Crosslinking experiment of GST-tagged recombinant proteins with 80% 18:1–16:0 PC and 20% 18:1–12:0 NBD–PE liposomes. GST-TRIM31-wild-type B-Box (GST-TRIM31-WT), GST-TRIM31-AXXA-mutated B-Box (GST-TRIM31-AXXA), or GST-glycine-exposed LC3 (GST-LC3-Gly) was incubated with liposomes, and then samples were analysed by SDS–PAGE. The LAS image analyser was used to detect NBD fluorescence (upper) while Coomassie blue staining was used to visualize protein levels (bottom). GST-LC3-Gly was used as a positive control. (**c**) Colocalization of wild-type (TRIM31-WT) or AXXA-mutated TRIM31-Myc (TRIM31-AXXA) with NBD–PE. HeLa cells were transfected with TRIM31-WT-Myc or TRIM31-AXXA-Myc, labelled with NBD–PE liposomes for 1 h, washed twice with PBS, and incubated for 1 h in complete media. Scale bars, 5 μm. Quantification is shown in the right graph (mean±s.d.). *P<0.01 (Student's *t*-test). (**d**) HeLa cells expressing mRFP-GFP-tagged wild-type TRIM31 (mRFP-GFP-TRIM31-WT) or AXXA-mutated TRIM31 (mRFP-GFP-TRIM31-AXXA) were treated with LPS (100 ng ml^−1^) for 4 h. Scale bar, 5 μm. (**e**) Palmitoylation is involved in forming a punctate structure of TRIM31. HeLa cells stably expressing TRIM31-Myc were left untreated or treated with 2-BP (20 μM) for 12 h. TRIM31-Myc was stained using anti-Myc antibody and analysed by IFA. Blue, DAPI. Scale bar, 5 μm. (**f**) Palmitoylation is essential for TRIM31–PE interaction. HeLa cells expressing TRIM31-Myc were left untreated or treated with 2-BP (20 μM) for 12 h and then labelled with 20% NBD–PE/80% POPC liposomes. TRIM31-Myc was immunostained with anti-Myc antibody and analysed by IFA. Blue, DAPI. Scale bar, 5 μm. (**g**) HeLa cells expressing mRFP-GFP-TRIM31 were incubated for 9 h in 2-BP (25 μM) and then stimulated with LPS (100 ng ml^−1^) for 3 h. Scale bar, 5 μm. All data are representative of at least two independent experiments.

TRIM31 is located within a double-membrane structure of the autophagosome even though it does not possess a membrane-spanning domain. Although we noted that TRIM31 interacts directly with PE, we also considered the involvement of other lipid modifications on TRIM31, such as myristoylation, prenylation and palmitoylation that acquire more sufficient hydrophobicity for membrane association and exclusive partitioning in the pellet fractions similar to LC3, Atg5, Atg7 or Atg12. In particular, recent studies showed that palmitoylation is critical for membrane association by immunity-related GTPase family M protein, which plays a role in autophagy and the reduction of intracellular bacteria^45^,^46^,^47^. We did not observe any well-defined consensus sequences for myristoylation or prenylation on TRIM31. Interestingly, bioinformatics analysis^48^,^49^,^50^ identified potential palmitoylated cysteine residues within the B-box region, which are highly conserved across various species (Fig. 5a).

Because palmitoylation occurs on cysteines, we constructed a point mutant by substituting cysteines with alanines. Then we assessed its puncta pattern in comparison to wild-type TRIM31. Notably, unlike wild-type TRIM31, this mutant was distributed in the cytosol and nucleus and we failed to observe puncta staining similar to TRIM31ΔBB (Supplementary Fig. 8a). We next examined whether TRIM31-AXXA is capable of crosslinking with PE. Strikingly, the NBD fluorescence signal of the TRIM31-AXXA mutant was considerably reduced at ∼80% (Fig. 5b, lane 5), indicating that cysteine residues within the TRIM31 B-box are critical for PE binding *in vitro*. Because this *in vitro* crosslinking experiment does not represent a direct involvement of palmitoylation on TRIM31-mediated autophagy process, we further explored whether TRIM31-AXXA mutant directly affects autophagy maturation in cells. Notably, this mutant failed to overlap with PE and punctate structures of LC3 in cells similar to TRIM31ΔBB (Fig. 5c; Supplementary Fig. 8b). In contrast to wild-type TRIM31, the expression of TRIM31-AXXA failed to detect a significant induction of LC3-II accumulation in HEK293T and HeLa cells (Supplementary Fig. 8c,d). Furthermore, we did not observe RFP signals from TRIM31-AXXA, indicating that these cysteine residues are critical to the induction of TRIM31-mediated autophagy (Fig. 5d). To examine whether the observed failure of the TRIM31-AXXA-PE interaction was not due to lack of access to PE as a result of disrupted subcellular targeting of the mutant to mitochondria, we performed subcellular fractionation for mitochondria and cytosol. Similar to wild-type TRIM31, TRIM31-AXXA was targeted to mitochondria (Supplementary Fig. 8e).

To further address whether palmitoylation is indeed involved in TRIM31-mediated autophagy activation, we exposed wild-type TRIM31-expressing cells to the palmitoylation-specific inhibitors, 2-bromopalmitic acid (2-BP) and tunicamycin, which are broadly used for inhibiting subcellular palmitoylation processes^51^,^52^,^53^,^54^. Markedly, wild-type TRIM31 failed to form a punctate structure in cells exposed to both inhibitors in a manner similar to TRIM31ΔBB or TRIM31-AXXA (Fig. 5e; Supplementary Fig. 8f). Moreover, 2-BP significantly affected TRIM31–PE binding and autolysosomal formation since a reduction in fluorescent signals of NBD–PE and RFP was observed (Fig. 5f,g). Taken together, these findings demonstrate that palmitoylation is essential for PE interaction with TRIM31, thus promoting autophagic membrane association and autolysosomal formation.

### Autophagy induced by TRIM31 inhibits invasive bacteria

**Figure 6:**
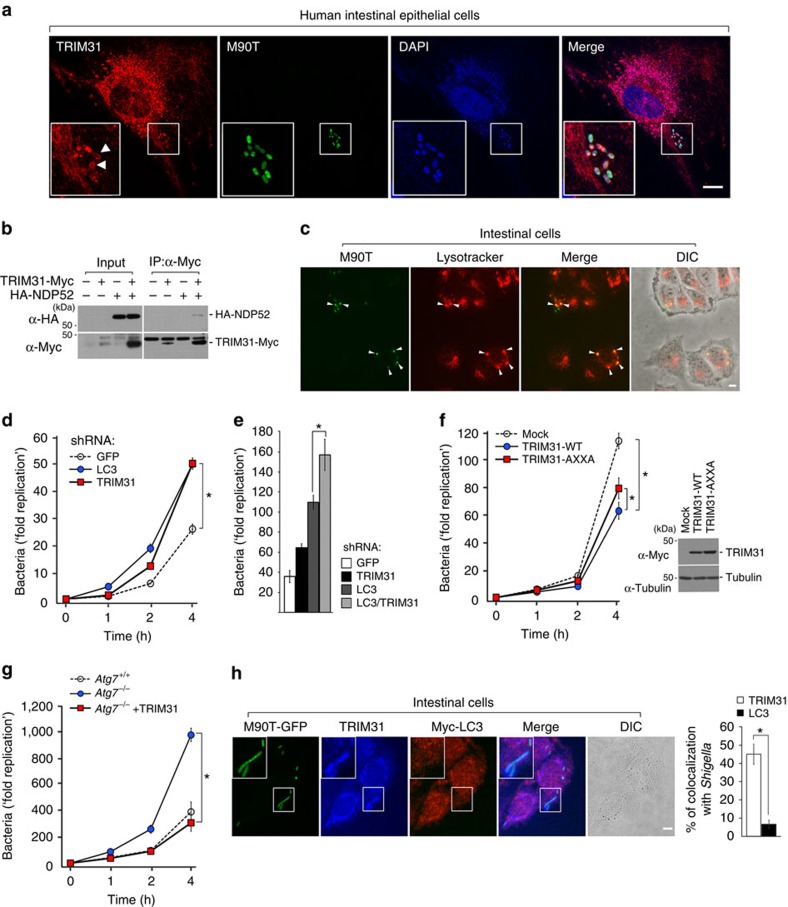
TRIM31-induced autophagy restricts growth of invasive bacteria in the intestinal epithelial cells. (**a**) Extensive colocalization of endogenous TRIM31 with *Shigella* and accumulation of TRIM31 around the surface of *Shigella*. Intestinal epithelial cells were infected with GFP-tagged *S. flexneri* M90T strain (green) and chased in complete media for 1 h with gentamicin (100 μg ml^−1^) to eliminate extracellular bacteria. Cells were immunostained with anti-TRIM31 antibody (red), followed by counterstaining with DAPI (blue). Scale bar, 5 μm. (**b**) Cell lysates from HEK293T cells expressing TRIM31-Myc or HA-NDP52 were lysed with 0.5% NP-40, immunoprecipitated with anti-Myc antibody, and then subjected to immunoblot analysis with anti-HA antibody. (**c**) Caco-2 cells were infected with GFP-tagged M90T and incubated with Lysotracker. Arrowheads indicate colocalization of intracellular *Shigella* with Lysotracker. DIC, differential interference contrast. Scale bar, 5 μm. (**d**) The bacteria recovery assay was performed in HT-29 cells stably expressing shRNA GFP (open circles), shRNA TRIM31 (closed red squares), or shRNA LC3 (closed blue circles). Cells were infected with M90T strain and chased in complete media with gentamicin (100 μg ml^−1^) for the indicated times. *P<0.01 (Student's *t*-test). (**e**) The bacterial recovery assay in HT-29 cells stably expressing shRNA against GFP, TRIM31, LC3 or LC3 and TRIM31. *P<0.01 (Student's *t*-test). (**f**) HeLa cells stably expressing control pLHCX vector (Mock, open circles), wild-type TRIM31-Myc (WT, closed blue circles), or AXXA-mutated TRIM31-Myc (AXXA, closed red squares) were infected with M90T-GFP. Expression of WT-TRIM31-Myc or AXXA-TRIM31-Myc was confirmed by immunoblot analysis using anti-Myc antibody. *P<0.01 (Student's *t*-test). (**g**) Wild-type or *Atg7*^−/−^ cells stably expressing mock or TRIM31-Myc were infected with M90T-GFP. *P<0.01 (Student's *t*-test). Open circles, *Atg7*^*+/+*^; closed blue circles, *Atg7*^−/−^; closed red squares, *Atg7*^−/−^+TRIM31. (**h**) Caco-2 cells stably expressing Myc-LC3 were infected with M90T-GFP (green) and incubated with media containing gentamicin (100 μg ml^−1^) for 1 h. Cells were immunostained with anti-TRIM31 (blue) and anti-Myc (red) antibodies. DIC, differential interference contrast. Scale bar, 5 μm. Right graph, quantification of TRIM31- or LC3-positive bacteria in Caco-2 cells. *P<0.01 (Student's *t*-test). All data are representative of at least three independent experiments (mean±s.d. in **d**–**h**).

Because TRIM31 can form autophagosomes/autolysosomes and LPS promotes TRIM31-mediated autolysosome formation, we speculated that intestine-specific TRIM31 may inhibit the survival of invasive bacteria by activating autophagy in the intestinal cells. *Shigella flexneri* are Gram-negative bacteria that are highly pathogenic for humans^55^,^56^,^57^. To investigate whether *Shigella* would be taken up into TRIM31-positive autophagosomes, we infected GFP-labelled pathogenic wild-type *S. flexneri* (M90T strain) into intestinal epithelial cells. We observed extensive colocalization of endogenous TRIM31 with *Shigella* and accumulation of TRIM31 around the surface of *Shigella* in intestinal and colonic cells (Fig. 6a; Supplementary Fig. 9a). In particular, NDP52 recognizes ubiquitin-coated *Salmonella* and *Shigella*, thus recruiting LC3 and activating autophagy^58^. To address whether this accumulation was due to the specific interaction of TRIM31 with the receptor recognizing ubiquitin-coated bacteria, we next examined the binding of TRIM31 with NDP52. Our data showed a considerable interaction between TRIM31 and NDP52 (Fig. 6b), suggesting that NDP52 recruits *Shigella* into TRIM31-positive autolysosomes.

These results led us to investigate whether TRIM31 is indeed required to restrict the growth of *Shigella* in epithelial cells. To test whether autophagy is involved in restricting *Shigella* growth, we first investigated the colocalization of *Shigella* with lysosomes and measured the rate of bacterial growth in chloroquine-treated cells. As expected, *Shigella* clearly colocalized with lysosomes similarly to TRIM31 and autolysosomal inhibition enhanced the proliferation of *Shigella* (Fig. 6c; Supplementary Fig. 9b). Notably, TRIM31 depletion also led to dramatic hyperproliferation of *Shigella*. In fact, the kinetics and magnitude of *Shigella* growth were very similar between HT-29 cells that were depleted of LC3 and TRIM31 (Fig. 6d). In addition, the *Shigella* survival rate in intestinal cells lacking both LC3 and TRIM31 was much higher than that in cells with depleted of LC3 or TRIM31 alone (Fig. 6e). Furthermore, expression of TRIM31-AXXA partially decreased *Shigella* growth (Fig. 6f). This partial effect might be due to endogenous LC3 expression in HeLa cells. These data suggest that TRIM31-induced autophagy is essential for restricting the growth of *Shigella* in epithelial cells.

We previously showed that TRIM31 induces LPS-induced alternative autolysosome formation (Fig. 3). In addition, TRIM31 clearly colocalized with PE even in HT-29 cells lacking Atg7, Beclin1 or LC3 (Fig. 4e; Supplementary Fig. 7b). Similarly, we observed extensive colocalization of endogenous TRIM31 with *Shigella* in HT-29 cells depleted of Atg7, Beclin1 or LC3 (Supplementary Fig. 9c). We thus sought to examine whether TRIM31-mediated autophagy promoted bacteria elimination via an alternative autophagy pathway. Because canonical autophagy is known to be essential for eliminating intracellular bacteria, when *Atg7*^−/−^ cells were infected with *Shigella, Atg7*-deficient cells failed to clear bacteria, thus severely increasing the bacterial load. Interestingly, introduction of TRIM31 into *Atg7*^−/−^ cells restored their ability to clear *Shigella* (Fig. 6g). Furthermore, a large amount of *Shigella* colocalized with TRIM31 rather than LC3 (Fig. 6h; Supplementary Fig. 9d). Taken together, TRIM31 induces alternative autophagy, which is essential for eliminating intracellular pathogenic *Shigella* in intestinal cells.

### Antibacterial effect requires control of TRIM31 expression

**Figure 7:**
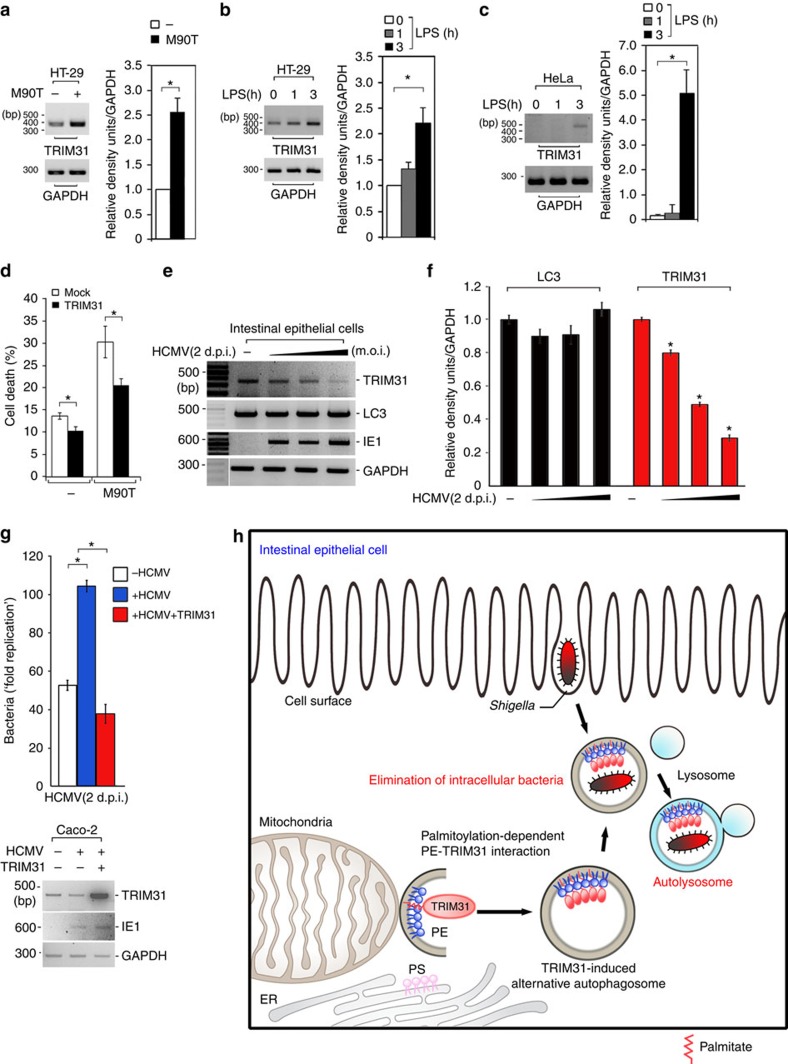
HCMV infection abrogates TRIM31 expression. (**a**) Left, HT-29 cells were infected with invasive *Shigella* M90T strain. TRIM31 mRNA levels were determined by RT-PCR. Right, Quantification of TRIM31 mRNA levels normalized against GAPDH, which was used as a loading control. *P<0.01 (Student's *t*-test). (**b**) HT-29 cells were treated with LPS (100 ng ml^−1^) for 0, 1 or 3 h, and then TRIM31 mRNA was measured by RT-PCR. *Right*, Quantification of TRIM31 mRNA levels normalized against GAPDH, which was used as a loading control. *P<0.01 (Student's *t*-test). (**c**) TRIM31 mRNA was measured by RT-PCR in HeLa cells treated with LPS (100 ng ml^−1^) for 0, 1 or 3 h. *P<0.01 (Student's *t*-test). (**d**) Cell death was assessed by propidium iodide (PI) staining of mock or TRIM31-Myc-expressing HeLa cells infected with M90T-GFP followed by flow cytometric analysis. Values represent the average of three independent experiments. *P<0.05 (Student's *t*-test). (**e**) HCMV infection down-regulates TRIM31 expression. Wild-type HCMV AD169 strain was infected into the intestinal epithelial cell line, Caco-2, for 48 h, and then HCMV immediate early gene (IE1), LC3, or TRIM31 mRNA was measured by RT-PCR. (**f**) Quantification of TRIM31 and LC3 mRNA levels normalized against GAPDH. *P<0.01 (Student's *t*-test). (**g**) HCMV-infected intestinal cells show a considerable increase in bacteria load. Intestinal cells were infected with HCMV for 48 h, followed by infection with *Shigella* prior to measuring the degree of bacterial growth. TRIM31 or IE1 mRNA levels were determined by RT-PCR. Values represent the average number of colonies from three independent experiments. *P<0.01 (Student's *t*-test). All data are representative of at least three independent experiments (mean±s.d. in **a**–**d**, **f**–**g**). (**h**) Proposed model of TRIM31-mediated alternative autophagy process in the intestinal epithelial cells.

Invasive bacteria activate intestinal epithelial autophagy^59^. Thus, we proposed that invasive bacteria might elicit a high level of TRIM31 expression, thus promoting intestinal autophagy to maintain immune homoeostasis. To explore this hypothesis, HT-29 cells were infected with *Shigella* and then TRIM31 expression was examined by reverse transcription (RT)-PCR. Remarkably, the invasive *Shigella* elicited enhanced TRIM31 gene expression (Fig. 7a). Since Toll-like receptor 4 (TLR4) recognizes LPS, which controls autophagy as a sensor^38^,^39^, we examined the effect of LPS on the activation of TRIM31 gene expression. TRIM31 mRNA was markedly increased in a time-dependent manner (Fig. 7b), suggesting that LPS-mediated TLR4 signalling is involved in eliciting TRIM31 gene expression to activate alternative autophagy and regulate intestinal homoeostasis.

Because TRIM31 is mainly expressed in the intestine, we did not observe TRIM31 expression in HeLa cells, which normally express LC3. Interestingly, when HeLa cells were treated with LPS, we clearly observed TRIM31 expression at 3 h post stimulation (Fig. 7c). Furthermore, we observed recruitment of *Shigella* into TRIM31-positive autophagosomes in HeLa cells similar to intestinal epithelial cells (Supplementary Fig. 9e). Additionally, since it is known that *Shigella* invades HeLa cells with high efficiency, bacteria-induced apoptosis readily occurs in a caspase-1-dependent manner^60^,^61^. Thus, we hypothesized that if *Shigella* is not efficiently eliminated by autophagy activation, HeLa cells may be more susceptible to apoptosis following robust bacterial growth. To test this, we evaluated the degree of apoptosis in *Shigella*-infected HeLa cells that expressed an empty vector or TRIM31 by using propidium iodide staining. Indeed, *Shigella* clearly induced apoptosis (Fig. 7d). Markedly, in TRIM31-overexpressing HeLa cells, the level of apoptosis was considerably decreased, indicating that a TRIM31-dependent alternative autophagy is essential to block disseminating intracellular *Shigella* in HeLa cells, thus protect against bacteria-induced apoptosis.

To understand the physiological relevance of human TRIM31-induced alternative autophagy in intestinal epithelial cells, we utilized a human cytomegalovirus (HCMV) system. Because HCMV can infect the gastrointestinal tract, the prevalence of active HCMV infection in the colon is significantly higher in patients with IBD relative to controls, suggesting that IBD severity correlates positively with HCMV infection^62^,^63^,^64^,^65^. However, it remains unknown how HCMV infection affects IBD severity. Since we previously showed an aberrant low-level expression of TRIM31 in patients with Crohn's disease (Fig. 1f–h), we speculated that HCMV infection affects TRIM31 expression in intestinal epithelial cells. We infected the intestinal epithelial cell line Caco-2 with the wild-type HCMV strain AD169 and then examined endogenous TRIM31 expression. Remarkably, HCMV infection unambiguously downregulated endogenous TRIM31 expression in a dose-dependent manner, but not LC3 (Fig. 7e,f). A recent study showed a significant mucosal HCMV viral load in IBD patients relative to control populations^66^. To further determine whether the proliferation of *Shigella* is affected by HCMV infection, intestinal cells were infected with HCMV, followed by infection with *Shigella* before measuring the degree of bacterial growth. Interestingly, HCMV-infected intestinal cells showed a significant increase in bacteria load (Fig. 7g). To determine whether this hyperproliferation of *Shigella* in HCMV-infected cells is due to HCMV-mediated TRIM31 downregulation, we introduced wild-type TRIM31 into HCMV-infected intestinal cells and examined *Shigella* growth. Surprisingly, introduction of TRIM31 markedly restored the clearance of intracellular *Shigella* (Fig. 7g). This is consistent with the observation that lack of TRIM31 disrupts autophagic elimination of intracellular *Shigella* (Fig. 6). These results demonstrate that HCMV leads to TRIM31 downregulation at a later time during HCMV infection, reflecting the functional relevance of HCMV infection on IBD severity.

## Discussion

Our findings suggest that the intestinal autophagy process is considerably more complicated than previously thought and involves an additional pathway that functions independently of canonical Atg-mediated autophagy. The intestinal epithelium is a highly specialized region that interfaces with a dense microbial community, including non-pathogenic and pathogenic bacteria. Thus, we hypothesize that this alternative autophagy pathway functions along with the Atg5/7-dependent canonical autophagy to both limit an overflow of commensal bacteria and eliminate invasive organisms. Here, we report that intestine-specific TRIM31 is able to interact with mitochondrial PE in both wild-type and *Atg7*^−/−^ cells, thereby activating autophagosome formation. TRIM31–PE binding induces an Atg5/7-independent alternative autophagy pathway, which can eliminate invasive pathogenic *Shigella* bacteria (Fig. 7h). Our data also indicate that TRIM31-mediated non-canonical autophagy in the intestine can compensate for the functional roles of canonical autophagy following the loss of Atg proteins. Although both processes lead to bacterial clearance in the intestine, their activities may be appropriately balanced by protein expression and downstream activation, and further, these pathways may function together to promote healthy gut homoeostasis.

As noted previously, because TRIM31 is preferentially colocalized with NBD–PE, we originally predicted that this protein is conjugated with PE *in vivo*. Unfortunately, we failed to observe TRIM31–PE conjugation *in vivo* following purification of TRIM31 and subsequent mass spectrometry. Similar to the LC3-II purification described by Kabeya *et al*.^29^, the absolute amount of TRIM31 may be difficult to determine due to rapid degradation of this protein via self-ubiquitination and autophagy-mediated lysosomal targeting (Supplementary Fig. 4). Furthermore, in the study by Ichimura *et al*.^33^, which first demonstrated that Atg8 is covalently conjugated to PE using mass spectrometry, the experiments were performed using yeast cells *in vitro*, rather than live cells *in vivo.* Based on both these reports and our trials, we may be confronted with a technical challenge in determining the absolute TRIM31 yield after purification or elution from the gels. Additionally, in experiments with TRIM31 mutants containing glycine residues available for covalent coupling with PE, the TRIM31 constructs were distributed throughout the nucleus or cytoplasm, and we failed to detect any punctate structures, similar to the pattern observed with the TRIM31ΔBB or TRIM31-AXXA mutants (Supplementary Fig. 6a–c). These results suggest that the TRIM31–PE interaction is mediated by glycine-independent covalent binding.

Our data further suggest that palmitoylation of TRIM31 is critical for this alternative autophagy pathway. Namely, we found a significant defect of punctate structure formation in cells exposed to palmitoylation-specific inhibitors, and observed a similar subcellular localization pattern in cells containing a TRIM31-AXXA mutant, in which cysteine residues predicted to function as potential palmitoylation sites were replaced with alanines. However, we have been unable to show a direct palmitoylation of TRIM31 at these cysteines. Of note, the B-box of MuRF1 coordinates two zinc ions through highly conserved cysteine residues^67^, assuming that these cysteines within the B-box would be inaccessible for palmitoylation. However, Gottlieb *et al*.^68^ suggested that the cysteine-rich domain of the DHHC3 protein contains tightly bound zinc ions, which is required for palmitoylation at the cysteine residues. Moreover, mutation of the DHHC motif cysteine abolishes detectable incorporation of palmitate and eliminates the steady state palmitoylation of DHHC3 palmitoyltransferases. Similarly, because TRIM31 consists of 12 cysteine residues in the RING finger and B-box domains, other cysteines might be involved in coordinating zinc ions, which may contribute to efficient palmitoylation at the cysteine residues within the TRIM31 B-box domain. Fairbank *et al*.^69^ also showed that the RING finger domain of the GP78 ubiquitin ligase contains a conserved series of cysteine residues and is palmitoylated at a specific subset of these residues, and this modification is critical for its subcellular distribution to the peripheral endoplasmic reticulum. In a similar context, it is possible that the RING finger of TRIM31 may also be involved in palmitoylation, which could have significant implications for TRIM31-mediated autophagy. In this regard, other cysteines in TRIM31 are also plausible candidates for possible palmitoylation, potentially functioning to promote interaction with PE and autophagy induction.

TLRs recognize different components from bacteria, fungi and viruses to promote various cytokines and chemokines that are critical for inducing innate and adaptive immune responses^70^. Because TLR4 is essential for LPS recognition, many adaptor proteins involved in activating this receptor could also be critical in LPS-induced TRIM31 gene expression and its modification. Particularly, TRIM31 is a member of RBCC proteins composed of RING finger, B-box and coiled-coil domain. Consistent with Sugiura and Miyamoto^21^, who reported that TRIM31 possesses E2-selective ubiquitin ligase activity, our results showed that TRIM31 regulates its expression levels via RING domain-dependent auto-ubiquitination. It is possible that LPS-mediated poly-ubiquitination of TRIM31 may occur at later times after autophagy activation, thus preventing excessive TRIM31-mediated alternative autophagy process and maintaining intestinal homoeostasis.

As noted previously, HCMV infection in the colon is considerably higher in patients with IBD relative to controls, indicating that IBD severity correlates positively with HCMV infection^62^,^63^,^64^,^65^. Based on our findings, HCMV targets TRIM31 downregulation, resulting in hyperproliferation of invasive bacteria. Thus, HCMV infection may be a risk factor for developing severe IBD. This is the first evidence for the molecular and physiological effect of HCMV on hindering host autophagy against pathogenic intestinal bacteria by targeting TRIM31.

## Methods

### DNA constructs

The human TRIM31 cDNA was purchased from Origene (#MHS6278-202756045; GE Healthcare, Marlborough, MA, USA) and subcloned into the pLHCX vector (Clontech, Mountain View, CA, USA) using the 5′-GCTAAGCTTGCCACCATGGCCAGTGGGCAGTTTGTGAA-3′ (HindIII-TRIM31; forward) and 5′-GCTAGATCTGCTTGAAGGAACCTTACAAAACCAAG-3′ (BglII-TRIM31; reverse) primers. All human TRIM31-related constructs were fused at the C terminus to Myc. N- or C-terminal deletion constructs tagged at the C terminus with Myc (ΔN-Myc (61–425) and ΔC-Myc (1–293)) were generated by PCR. The B-Box (ΔBB-Myc) or RING (ΔRING-Myc) deletion construct was generated by overlap extension PCR with the indicated primer pairs. All primers are listed in Supplementary Table 1. Human NDP52 cDNA was isolated by PCR using the cDNA library from HeLa cells with the following primers: NDP52 forward, 5′-AATGTTAACGGATCCATGGAGGAGACCATCAAAGAT-3′; reverse, 5′- AATATCGATCTCGAGTCAGAGAGAGTGGCAGAAC-3′. All constructs were cloned into the retroviral pLHCX (hygro) vector (Clontech, Mountain View, CA, USA) and verified by sequencing. EGFP-LC3 (Addgene plasmid #11546) or mRFP-GFP-LC3 (Addgene plasmid #21074) was kindly provided by Dr Karla Kirkegaard (Stanford University, Palo Alto, CA, USA) or Dr Tamotsu Yoshimori (Osaka University, Japan), respectively. HA-Atg12 (Addgene plasmid #22950) and Atg5-HA (Addgene plasmid #22948) were kindly supplied by Dr Noboru Mizushima (University of Tokyo, Japan), and Myc-Atg7 (Addgene plasmid #24921) was kindly provided by Dr Toren Finkel (NIH, USA).

### Reagents

Antibodies to anti-TOM20 (sc-11415, 1:2,000) and anti-GST (sc-459, 1:1,500) were purchased from Santa Cruz (Santa Cruz Biotechnology, Santa Cruz, CA, USA). Anti-LC3B (#2775, 1:1,000) and anti-Myc (#2276, 1:2,000) antibodies were obtained from Cell Signaling Technology (Danvers, MA, USA). Anti-TRIM31 (H00011074-B01, 1:500 and 12543-1-AP, 1:2,000) antibody was purchased from Abnova (Taipei City, Taiwan) and Proteintech (Rosemont, IL, USA), and mouse monoclonal anti-Tubulin (#G094,1:2,000) antibody was obtained from Applied Biological Materials Inc. (Richmond, Canada). Anti-p62 (#GP62-C, 1:2,000) antibody was purchased from Progen Biotechnik (Heidelberg, Germany). LysoTracker Red (#L-7528) and AlexaFluor 350-, AlexaFluor 488-, and AlexaFluor 568-conjugated antibodies were obtained from Life Technologies (Carlsbad, CA, USA). Lipids, namely, 18:1–16:0 PC (1-oleoyl-2-palmitoyl-*sn*-glycero-3-phosphocholine, #850475), NBD-Sphingosine omega ((7-nitro-2-1,3-benzoxadiazol-4-yl)(2S,3R,4E)-2-aminooctadec-4-ene-1,3-diol, #810205), and 18:1–12:0 NBD–PE (1-oleoyl-2-{12-[(7-nitro-2-1,3-benzoxadiazol-4-yl)amino]dodecanoyl}-*sn*-glycero-3-phosphoethanolamine, #810156) were purchased from Avanti Polar Lipids (Alabaster, AL, USA). Gentamicin sulfate salt (#G1264) and 2-Bromopalmitic acid (#238422) were obtained from Sigma-Aldrich (St Louis, MO, USA). The *S. flexneri* strain, invasive M90T-GFP was kindly provided by Dr Dong Wook Kim (Hanyang University, South Korea).

### Cell culture and transfection

Human embryonic kidney (HEK) 293T cells (CRL-3216, ATCC, Manassas, VA, USA), human foreskin fibroblast (HFF) cells (SCRC-1041, ATCC), the breast cancer cell line MCF-7 (HTB-22, ATCC), cervical cancer cell line HeLa (CCL-2, ATCC), kidney carcinoma cell line Caki-2 (HTB-47, ATCC), trophoblast cell line JEG-3 (HTB-36, ATCC), gastric cancer cell line AGS (CRL-1739, ATCC), lung cancer cell line A549 (CCL-185, ATCC), colonic epithelial cell line SW480 (CCL-228, ATCC), intestinal epithelial cell line Caco-2 (HTB-37, ATCC), and MEFs (SCRC-1008, ATCC) were cultured in Dulbecco's modified Eagle medium (DMEM) supplemented with 10% heat-inactivated fetal bovine serum (HyClone, Logan, UT, USA) and penicillin/streptomycin (HyClone). The colonic epithelial cell line HT-29 (HTB-38, ATCC) and monocytic cell line U937 (CRL-1593.2, ATCC) were cultured in RPMI containing 10% heat-inactivated fetal bovine serum and penicillin/streptomycin. Normal intestinal epithelial cells, FHs74 (CCL-241, ATCC) were cultured in Hybri-Care Medium ATCC 46-X supplemented with 30 ng ml^−1^ epidermal growth factor (EGF) and 10% fetal bovine serum. Tet-regulated *Atg5*^−/−^ or *Atg7*^−/−^ MEF cells were kindly provided by Dr Mizushima or Dr Komatsu, respectively. Tet-regulated *Atg5*^−/−^ MEF cells were maintained in complete DMEM with or without 10 ng  ml^−1^ doxycycline hyclate (Sigma-Aldrich). Cells were transfected using Omicsfect (OmicsBio, Taipei City, Taiwan) in serum-free and antibiotic-free DMEM for 30–36 h. All cells were grown at 37 °C in humidified air with 5% CO_2_.

### Retroviral transduction and RNAi production

HEK293T cells were transfected with plasmids encoding VSV-G and Gag-Pol, as well as shRNA for GFP (control), TRIM31, LC3B, Atg7 or Beclin1. At 48 h post-transfection, media containing viral particles were collected, filtered through a 0.45-μm membrane, and then incubated with cells for 24 h. Cells were selected with puromycin. The shRNA sequences were subcloned into the pSUPER retroviral vector (Oligoengine, Seattle, WA, USA). All oligomer sequences are listed in Supplementary Table 1.

### RT-PCR

Total cellular RNA was isolated using an RNA prep kit (GeneAll, Seoul, South Korea). RNA (0.5 μg) was reverse transcribed for 1 h with oligo dT at 42 °C using Moloney Murine Leukemia Virus (M-MLV) reverse transcriptase (Enzynomics, Daejeon, South Korea). All primer sequences are listed in Supplementary Table 1. Detailed CD patient information is included in Supplementary Table 2

### NBD–PE formulation and labelling

To prepare liposomes, 80% PC and 20% NBD–PE were mixed from stocks dissolved in chloroform. After transfer to a glass tube, the chloroform solvent was removed by rotary evaporation for 1 h. Samples were dried further in a desiccator under vacuum for 12 h. The resulting lipid film was suspended in a buffer (25 mM Tris-HCl, pH 7.5, 137 mM NaCl, 2.7 mM KCl) at a final concentration of 1 mM phospholipids by vortexing at room temperature. Samples were then subjected to sonication for 5 min to obtain small unilamellar liposomes. Samples were added to cells at a 0.15 mM concentration for 1–2 h to label the cells. Cells were washed with warm PBS and incubated in DMEM or RPMI supplemented with 10% FBS/penicillin/streptomycin for 1–2 h. After washing with PBS, cells were fixed in 3.7% formaldehyde, permeabilized with 0.1% Triton X-100 for 1 min, and incubated with primary antibodies overnight at 4 °C. Cells were washed three times with PBS and incubated with the appropriate AlexaFluor 568-conjugated secondary antibody (1:200, Life Technologies). DAPI (4′, 6-diamidino-2-phenylindole; Sigma-Aldrich) was used as a nuclear counterstain. Images were collected using a confocal microscope (LSM 700, Carl Zeiss, Jena, Germany).

### Coimmunoprecipitation and immunoblotting

Cells were lysed with 0.5% NP-40 in PBS supplemented with protease inhibitors for 1 h at 4 °C. After pre-clearing lysates with protein G-Sepharose (Sigma-Aldrich), primary antibodies and then protein G-Sepharose were added to the supernatants and incubated at 4 °C. The protein G-Sepharose beads were washed three times with 0.1% NP-40/PBS. Proteins were eluted from the beads by boiling in 2 × Denature buffer (50 mM Tris-HCl, pH 6.8, 2% SDS, 5% β-mercaptoethanol). Proteins were separated by SDS–polyacrylamide gel electrophoresis (SDS–PAGE), transferred onto a polyvinyl difluoride (PVDF) membrane (Millipore, Billerica, MA, USA), and probed with the appropriate antibodies overnight at 4 °C. Membranes were washed three times with PBS containing 0.1% Tween-20 and incubated with horseradish peroxidase-conjugated secondary antibodies for 1 h at room temperature. The immunoblots were visualized with enhanced chemiluminescence detection reagents (Advansta, Menlo Park, CA, USA). Uncropped images of western blots are shown in Supplementary Fig. 10.

### Infection and bacterial assays

*S. flexneri* M90T-GFP was grown overnight in LB broth and subcultured (1:10) for 6 h before infection. After collection by centrifugation, bacteria were resuspended in warm PBS using the same volume of LB broth. Then, the bacterial solution was added to cells in 24-well plates. Plates were centrifuged for 10 min at 670*g*. After incubation at 37 °C for 20 min, cells were washed twice with warm PBS and then incubated in 100 μg ml^−1^ gentamicin for the duration of the experiment to count intracellular bacteria. Cells from triplicate wells were lysed in PBS containing 0.1% Triton X-100 and plated in duplicate onto agar plates containing ampicillin. For the immunofluorescence assays, M90T-GFP-infected cells were washed twice with warm PBS and incubated in 100 μg ml^−1^ gentamicin for 30 min. Lysosomes were labelled by incubating cells with Lysotracker Red for 30 min during their incubation with bacteria.

### Immunohistochemistry

Formalin-fixed paraffin-embedded tissue sections (adjacent normal tissues from cancer patients and inflamed tissues from Crohn's patients operated for colon) were stained for anti-TRIM31 antibody (1:100) and counterstained with hematoxylin. To quantify TRIM31 expression, we studied five randomly selected 0.159-mm^2^ fields for each sample at × 100 magnification and scored range from 0 to 3. Detailed CD patient information is included in Supplementary Table 2.

### Immunoelectron microscopy

Cells were fixed in a mixture of 2% formaldehyde and 0.1% glutaraldehyde in 0.1 M cacodylate buffer (pH 7.2) for 30 min at 37 °C initially, rinsed with buffer, enclosed in 15% gelatin, infiltrated stepwise with 0.6, 1.2 and 2.3 M sucrose, and then frozen in liquid nitrogen. Frozen ultrathin sections were prepared at −110 °C according to Tokuyasu. Briefly, immuno-gold staining using mouse anti-Myc antibody (1:10) was followed by several rinses in PBS and incubation with 12-nm gold-labelled donkey anti-mouse IgG (715-205-150, Jackson Immunoresearch Laboratories, Inc., Bar Harbor, ME, USA) as described by Roth *et al*. Ultrathin frozen sections were observed with a Hitachi H-7650 electron microscope at 80 kV. The images were recorded using an 11-megapixel CCD XR611-M digital camera (Advanced Microscopy Techniques, Woburn, MA, USA).

### HCMV infection

The AD169 strain of HCMV was used in this study. Virus was maintained in HFF monolayers. To infect Caco-2 cells with AD169, monolayers were plated in 24-well plates and media was replaced with fresh DMEM before infection. HCMV infection was enhanced by centrifugation at 940*g* for 45 min. After centrifugation, cells were incubated for 3 h at 37 °C in an atmosphere of 5% CO_2_, washed two times with PBS, and then supplied with fresh complete media.

### Production of GST fusion proteins

*Escherichia coli* BL21 (DE3) expressing the appropriate GST fusion protein was cultured in LB broth. The proteins were induced with 0.5 mM isopropyl-β-D-thiogalactoside at 28 °C for 5 h, followed by centrifugation, and resuspended in PBS (137 mM NaCl, 2.7 mM KCl, 10 mM Na_2_HPO_4_, 2 mM KH_2_PO_4_) with EDTA-free protease inhibitors. Cells were sonicated and then centrifuged for 20 min at 4 °C. Extracts were incubated with 80 μl GST-bind agarose resin for 2 h at 4 °C. After washing with PBS, the beads with the GST fusion proteins were eluted with 20 mM GSH in PBS.

### Crosslinking experiment

Crosslinking reactions were carried out as described previously^42^. Briefly, liposomes consisting of 20% NBD–PE/80% PC or 20%NBP-Sphingosine/80%POPC and crosslinker N-α-maleimidoacet-oxysuccinimide ester (AMAS) dissolved in DMSO were incubated at 30 °C for 30 min in reaction buffer (100 mM HEPES/NaOH (pH 7.3), 150 mM NaCl) and further incubated for 15 min after the addition of 1 M glycine in reaction buffer (final concentration: 48 mM). GST fusion recombinant proteins (final concentration: 93.75 mg ml^−1^) were added to the reaction mixtures, which were then incubated at 30 °C for 30 min. Crosslinking was terminated by the addition of 5 × SDS loading buffer. Samples were resolved by SDS–PAGE and analysed by an LAS image analyser for the detection of NBD fluorescence (excitation 460 nm/emission 515 nm) followed by Coommassie Brilliant Blue staining.

### Protein-lipid overlay assay

Lipids were solubilized in a solution of MeOH:CHCl_3_:H_2_O (2:1:0.8), spotted onto Hybond-C extra Nitrocellulose (GE Healthcare), and allowed to dry. The nitrocellulose was then blocked with 1% fat-free skim milk in PBS-T for 1 h and incubated overnight at 4 °C in the PBS-T solution containing purified GST-recombinant protein (2 μg ml^−1^). The strips were washed 3 times with PBS-T and incubated with anti-GST antibody for 1 h. The membrane was washed as before prior to incubation with horseradish peroxidase-conjugated-anti-rabbit Ig antibody. Finally, the membrane was washed three times with PBS-T and proteins associated with the phospholipids were visualized using enhanced chemiluminescence.

### Mitochondria purification

Mitochondrial and cytosolic fractions were obtained by using the Mitochondria Isolation Kit for Cultured Cells (#89874, Thermo Scientific, Rockford, IL, USA). Briefly, 7 × 10^6^ cells were pelleted by centrifugation at 300*g* for 2 min and mitochondria isolation reagent A was added to the pellet. Cells were vortexed for 5 s and then incubated on ice for 2 min. Mitochondria isolation reagent B was added, followed by vortexing for 5 s. Tubes were incubated on ice for 5 min and vortexed every 1 min. Mitochondria isolation reagent C was added, and the tubes were inverted several times. The supernatant obtained by centrifugation at 700*g* for 10 min at 4 °C was transferred to a new tube and centrifuged at 3,000*g* for 15 min. Mitochondria isolation reagent C was added to the pellet and centrifuged at 16,000*g* for 5 min. The pellet containing the mitochondrial fraction was resuspended in SDS loading buffer and analysed by immunoblot analysis. For alkali extraction, mitochondrial pellets were resuspended in a buffer (20 mM Hepes, 0.05 mM EDTA, 2 mM MgCl_2_, Complete Mini Protease Inhibitor Cocktail, pH 7.4). The same volume of 0.2 M NaCl or 0.2 M Na_2_CO_3_ was added, and then incubation was continued for 30 min on ice. Pellets were recovered by centrifugation (100,000*g*, 30 min, 4 °C), and both pellet and supernatant fractions were analysed by western blotting.

### Statistical analysis

All experiments were repeated at least three times with consistent results. Data are presented as mean and s.d. (as noted in figure legends). Statistical differences between two means were evaluated with the two-tailed unpaired Student's *t*-test. Differences with *P* values below 0.05 were considered significant. All data were normally distributed and the variances were similar between the groups being statistically compared. Sample size was based on previous experience with experimental variability, and no statistical method was used to predetermine sample sizes. No samples were excluded from the analysis. The experiments were not randomized. The investigators were not blinded to allocation during experiments or outcome assessment.

### Data availability

The authors declare that the data supporting the findings of this study are available within the article and its Supplementary Information files, or from the corresponding authors upon request.

## Additional information

**How to cite this article:** Ra, E. A. *et al*. TRIM31 promotes Atg5/Atg7-independent autophagy in intestinal cells. *Nat. Commun.* 7:11726 doi: 10.1038/ncomms11726 (2016).

## Supplementary Material

Supplementary Information Supplementary Figures 1-10, Supplementary Tables 1-2
