# Supplementary material for: TRIM31 promotes Atg5/Atg7-independent autophagy in intestinal cells
Source: Nat Commun. 2016 May 24;7:11726. doi: 10.1038/ncomms11726 (PMC4890305; doi:10.1038/ncomms11726)
Supplement: Supplementary Information — Supplementary Figures 1-10, Supplementary Tables 1-2 [file ncomms11726-s1.pdf]

Supplementary Figure 1. Ra et al.

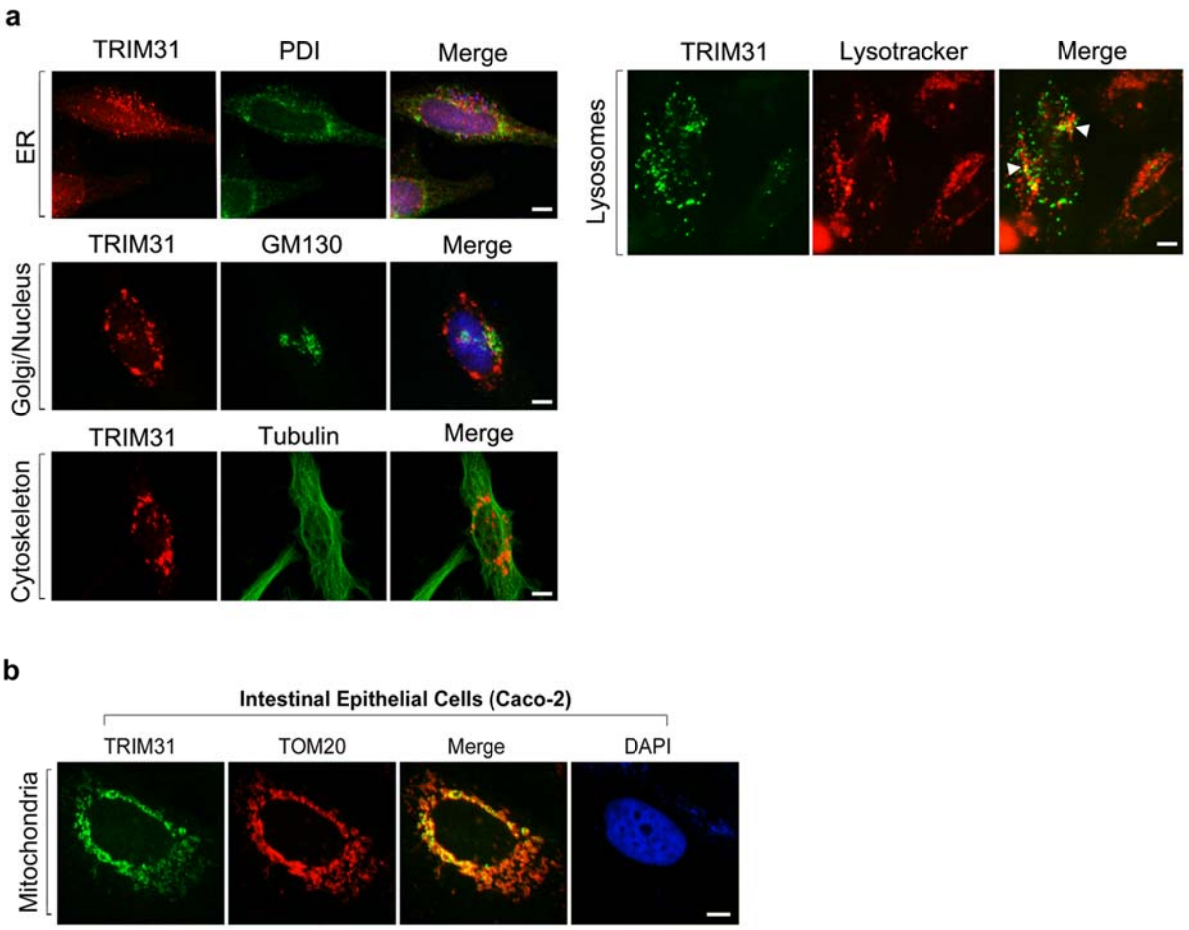

Supplementary Figure 1. TRIM31 is colocalized with lysosomes and mitochondria. (**a**) HeLa cells expressing TRIM31-Myc were immunostained with anti-Myc (TRIM31), anti-PDI (ER), anti-GM130 (Golgi), anti-Tubulin (cytoskeleton), and DAPI (nucleus). HeLa cells also transfected with vectors expressing GFP-TRIM31-Myc were treated with chloroquine (5 µM) for obtaining a clear image of lysosomes and stained with lysotracker (lysosomes). Cells were visualized by immunofluorescence assay. Scale bars, 5 µm. **(b)** TRIM31 is enriched in the mitochondria of intestinal cells (Caco-2). Intestinal epithelial cells were immunostained with anti-TRIM31 and anti-TOM20 (mitochondria) antibodies. All data are representative of at least two independent experiments.

Supplementary Figure 2. Ra et al.

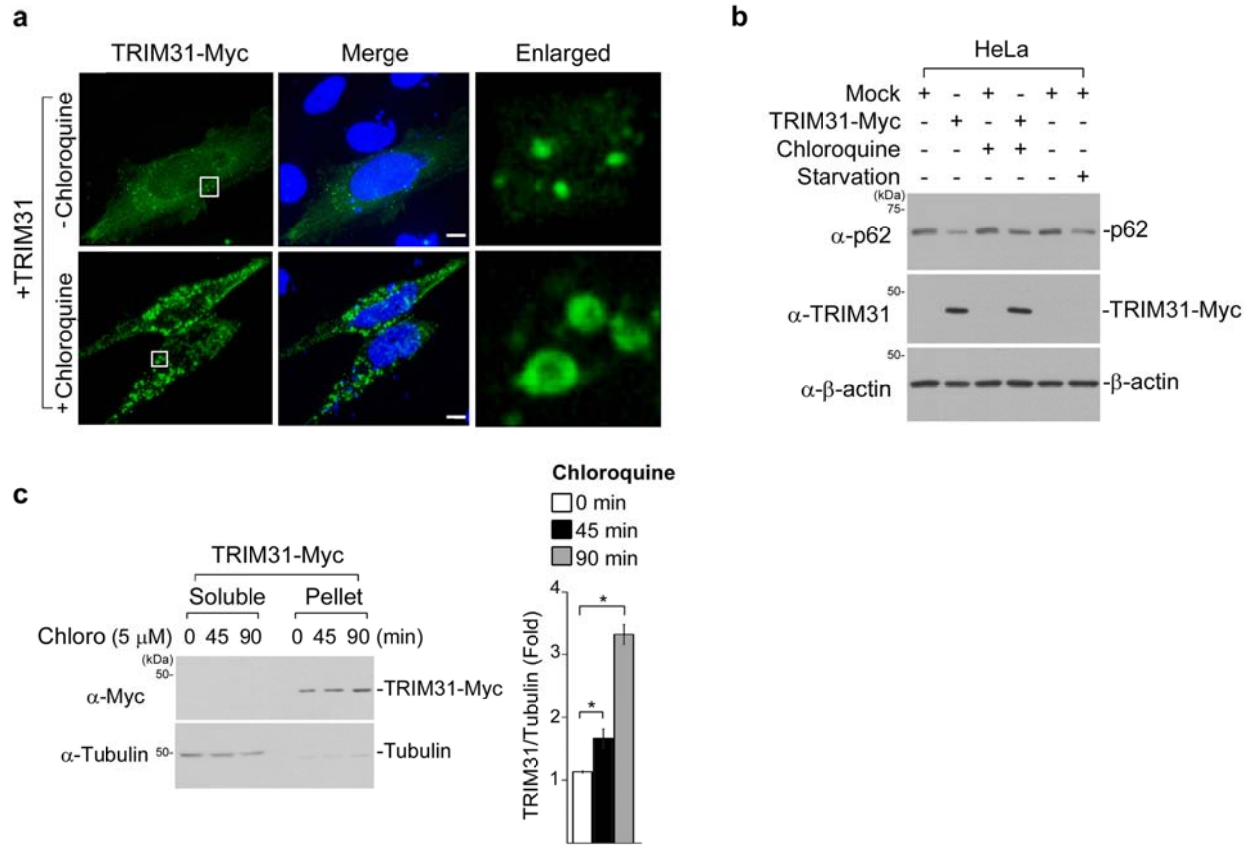

Supplementary Figure 2. TRIM31 induces autophagy-like vesicle formation. (a) HeLa cells transfected with vectors expressing TRIM31-Myc were left untreated or treated with chloroquine (5 μM) for 6 h, immunostained with anti-Myc (green) antibody, and visualized by confocal microscopy. Blue, DAPI. Scale bars, 5 μm. **(b)** HeLa cells were transfected with plasmids expressing empty vector or TRIM31-Myc and then incubated with chloroquine (5 μM) for 4 h or serum-free DMEM for 6 h. Endogenous p62 expression levels were determined by immunoblotting with anti-p62 antibody. **(c)** HeLa cells transiently expressing TRIM31-Myc were lysed with 0.5% NP-40 and separated into soluble and pellet fractions by centrifugation. Samples were subjected to SDS–PAGE and detected with primary antibodies as indicated. Quantification of TRIM31 protein levels as determined by scanning densitometry were normalized to tubulin in the *right graph*. *$P<0.01$ (Student's *t*-test). All data are representative of at least three independent experiments (mean ± s.d. in **c**).

Supplementary Figure 3. Ra et al.

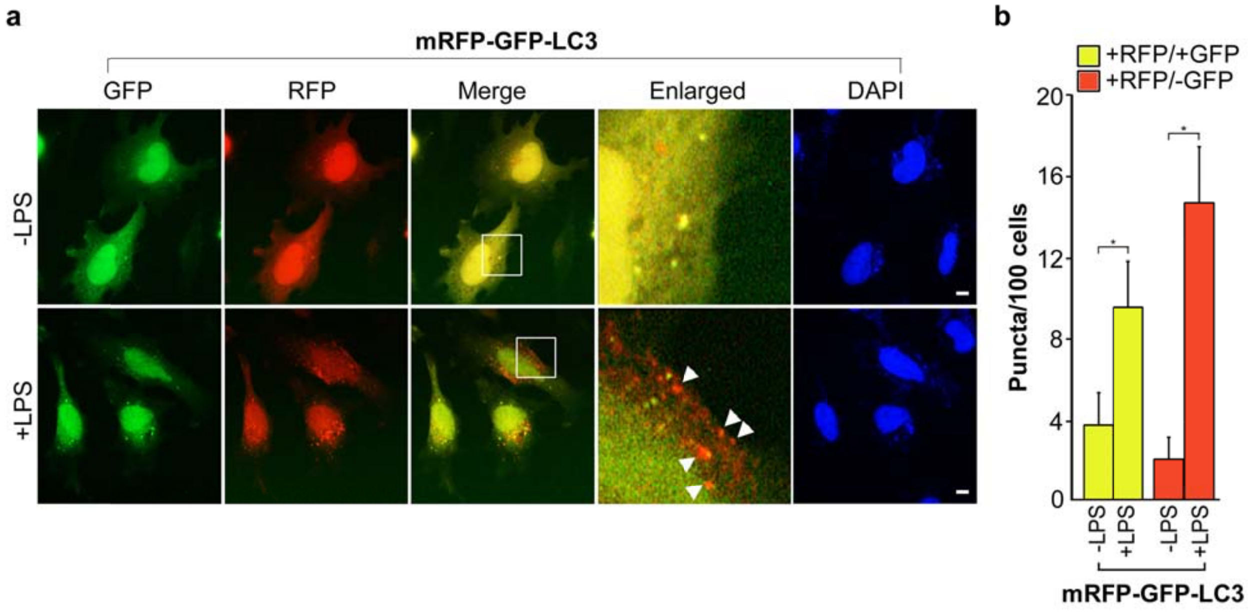

Supplementary Figure 3. LPS induces autolysosome formation. (**a**) HeLa cells expressing mRFP-GFP-LC3 were left untreated or treated with LPS (100 ng per ml) for 4 h. Blue, DAPI. Scale bars, 5 μm. Quantification for yellow or red punta is shown in the graph, **(b)**. *P<0.01 (Student's t-test). All data are representative of at least three independent experiments (mean ± s.d. in **b**).

Supplementary Figure 4. Ra et al.

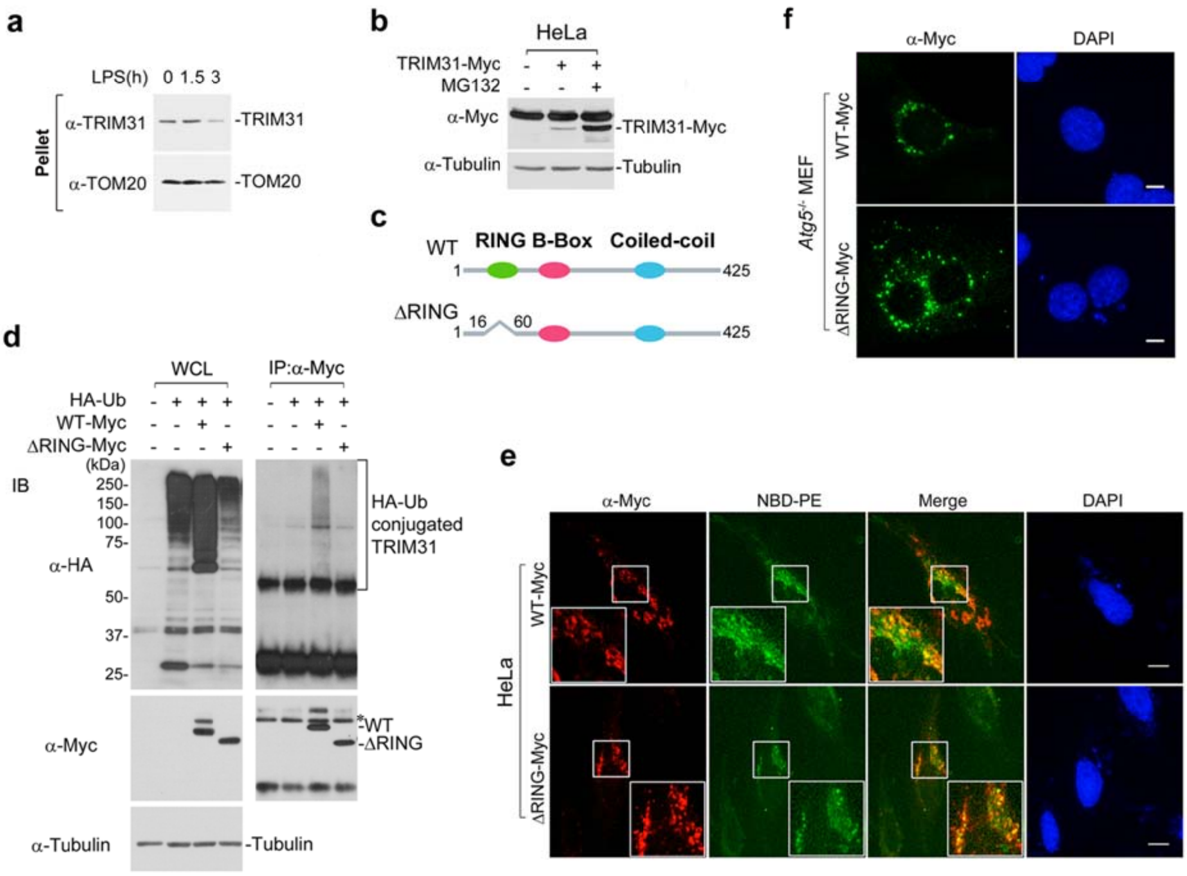

Supplementary Figure 4. TRIM31 is degraded in a proteasome-dependent manner. (a) HeLa cells transiently expressing TRIM31-Myc were treated for 1.5 h or 3 h with LPS, lysed with 0.5% NP-40, and separated into soluble and pellet fractions by centrifugation. Samples were subjected to SDS–PAGE and detected with primary antibodies as indicated. (**b**) HeLa cells transiently expressing TRIM31-Myc were left untreated or treated with MG132 (20 μM) for 4 h. **(c)** Schematic diagram of human TRIM31 and RING deletion mutants. The RING, B-box, and coiled-coil domains are depicted in green, pink, and blue, respectively. **(d)** Immunoblot analysis of the ubiquitination of wild-type TRIM31 (WT-Myc) or RING deletion mutant of TRIM31 (ΔRING-Myc) in HEK293T cells. Cells were transfected with WT-Myc, ΔRING-Myc, and HA-tagged ubiquitin (HA-Ub) as indicated. Ubiquitination levels of TRIM31 were determined by anti-HA antibody after immunoprecipitation with anti-Myc antibody. **(e)** RING deletion mutant of TRIM31 colocalizes with NBD-PE puncta. HeLa cells transiently expressing wild type TRIM31 (WT-Myc) or RING deletion mutant (ΔRING-Myc) were labeled with liposomes containing 20% NBD-PE and 80% POPC, followed by immunostaining with anti-Myc antibody. Scale bars, 5 μm. **(f)** *Atg5*$^{-/-}$ MEFs stably expressing wild type TRIM31 (WT-Myc) or RING deletion mutant (ΔRING-Myc) were stained using anti-Myc antibody and analyzed by immunofluorescence assay. Scale bars, 5 μm. All data are representative of at least two independent experiments.

Supplementary Figure 5. Ra et al.

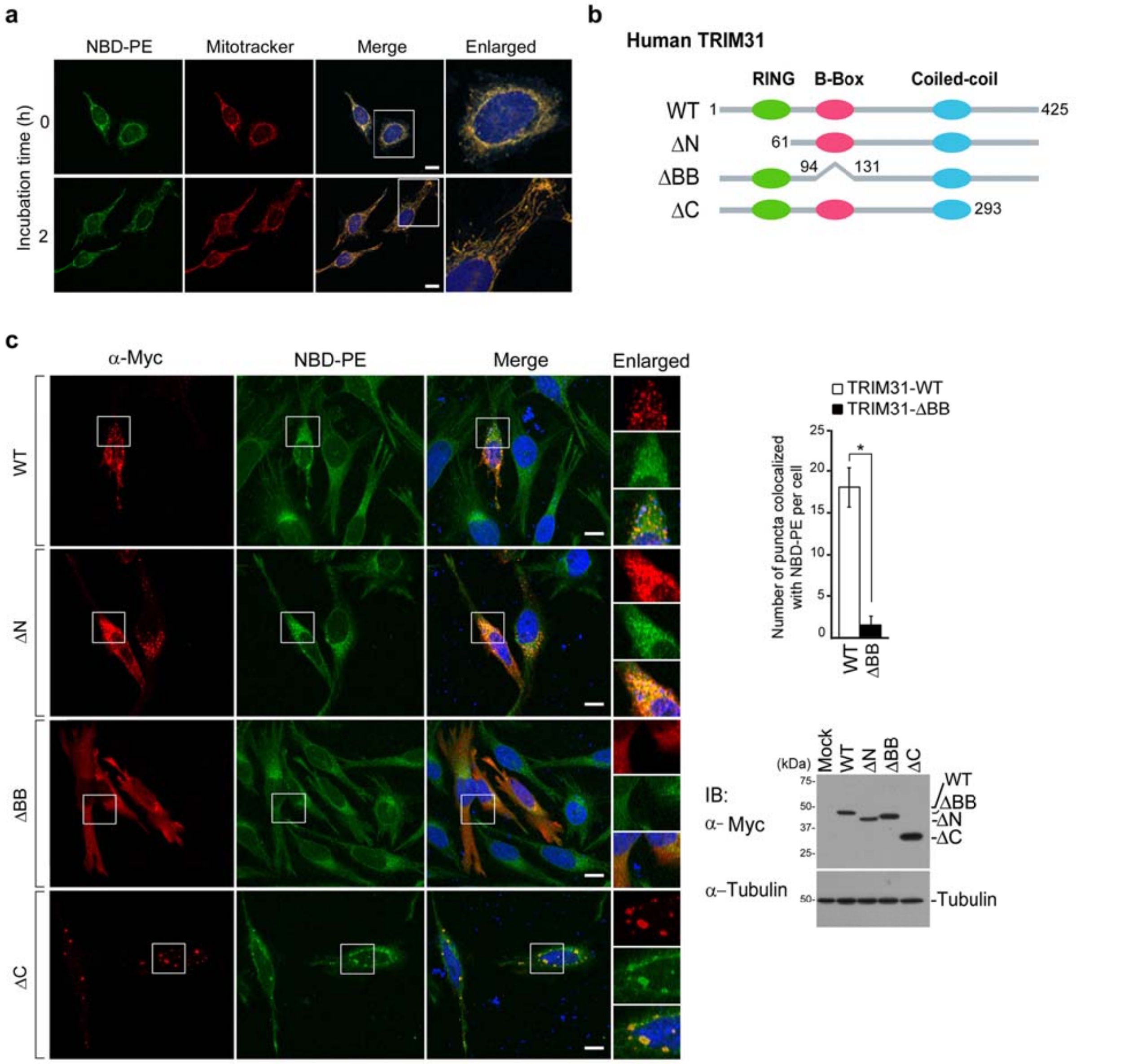

Supplementary Figure 5. The B-box domain of TRIM31 is required for PE interaction. (**a**) Time course following loading exogenous NBD-PE/POPC liposomes. Cells were exposed to liposomes, washed with PBS, and maintained in complete media for 0 or 2 h. MitoTracker (red) and NBD-PE (green) confirmed the accumulation of NBD-PE in mitochondria at 2 h after liposome loading. Blue, DAPI. Scale Bar, 5 μm. (**b**) A schematic diagram of human TRIM31 and the corresponding mutants. The RING, B-box, and coiled-coil domains are depicted in green, pink, and blue, respectively. Myc-tagged TRIM31 lacking the N-terminus region contained RING domain (TRIM31ΔN), B-box (TRIM31ΔBB), or the C-terminus region (TRIM31ΔC) (**c**) *left*, Myc-tagged wild-type TRIM31 (WT) and the corresponding deletion mutants (ΔN, ΔBB, ΔC-term) were individually transfected into HeLa cells, and then labeled with NBD-PE using NBD-PE/POPC liposomes. Cells were fixed and immunostained with anti-Myc antibody. Scale bars, 5 μm. Quantification for TRIM31-PE interaction is shown in the right graph. $*P<0.01$ (Student's *t*-test). *Right bottom panels*, expression of Myc-tagged wild-type TRIM31 and the corresponding deletion mutants were determined by immunoblot analysis. All data are representative of at least three independent experiments (mean ± s.d. in **c**).

Supplementary Figure 6. Ra et al.

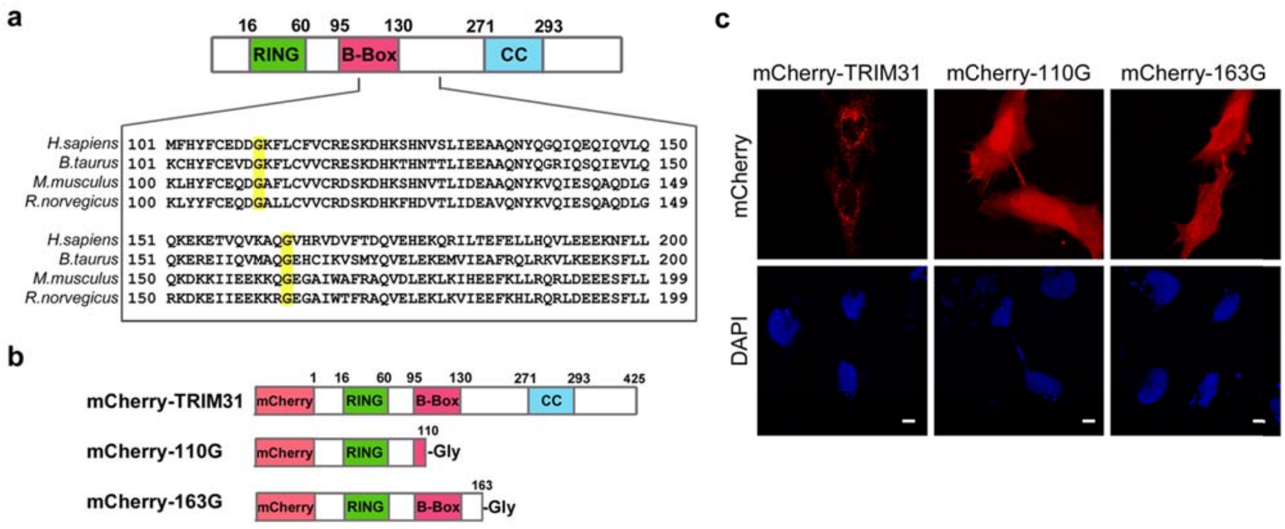

Supplementary Figure 6. Exposed glycine 110 and 163 of TRIM31 is not conjugated to phosphatidylethanolamine. (a) A schematic diagram of TRIM31 and multiple amino acid sequence alignment from residue 101 to 200. Yellow boxes indicate conserved glycine residues, Gly110 and Gly163. **(b)** Schematic representation of mCherry-tagged wild-type and C-terminal glycine-exposed TRIM31. Conserved glycine 110 and 163 are exposed at the C-terminus. **(c)** HeLa cells transiently expressing mCherry-TRIM31, mCherry-110G, and mCherry-163G were fixed and analyzed by immunofluorescence assay. Blue, DAPI. Scale Bar, 5 μm. All data are representative of at least three independent experiments.

Supplementary Figure 7. Ra et al.

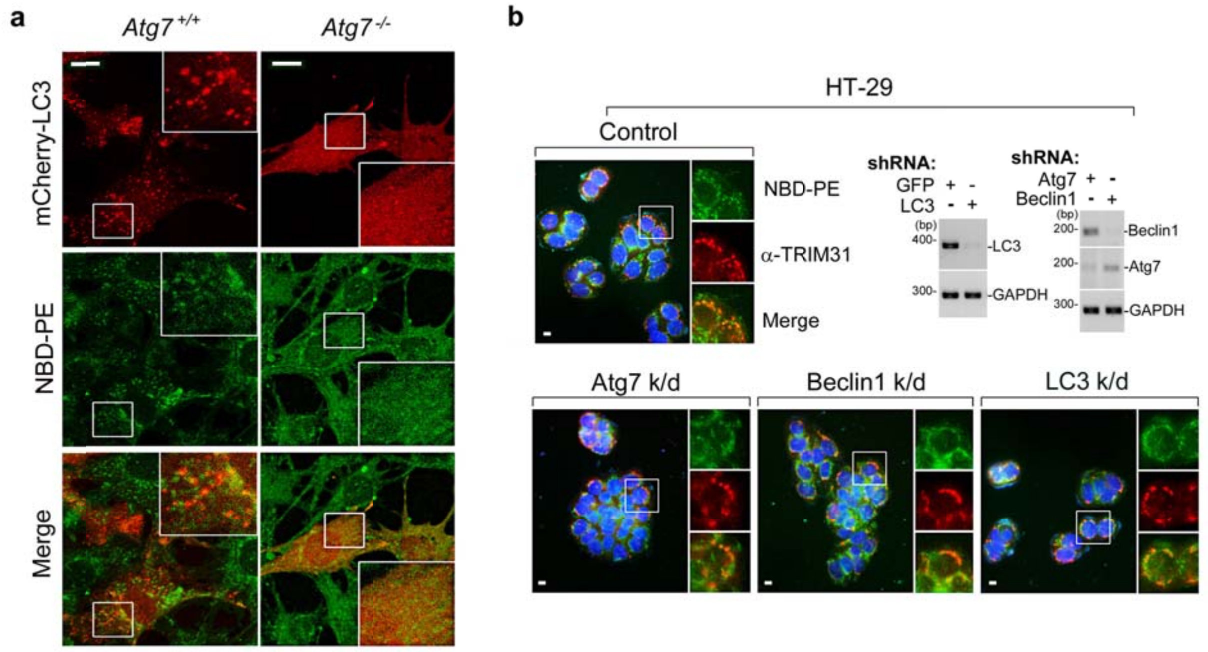

Supplementary Figure 7. TRIM31-PE interaction occurs via an alternative autophagy process. (a) Wild-type or *Atg7*$^{-/-}$ MEF cells transiently expressing mCherry-LC3 (red) were labeled with NBD-PE (green) for 1 h. Colocalization of mCherry-LC3 with NBD-PE was determined using confocal microscopy. Scale bar, 5 µm. **(b)** HT-29 cells stably expressing shRNA for Atg7, Beclin1, or LC3 were labeled with NBD-PE (green) using 20% NBD-PE/80% POPC liposomes for 1 h, fixed in 3.7% formaldehyde, and immunostained with anti-TRIM31 antibody. Blue, DAPI. Scale bars, 5 µm. Depletion of Atg7, Beclin1, or LC3 was confirmed by RT-PCR using the indicated primer pairs. All data are representative of at least three independent experiments.

Supplementary Figure 8. Ra et al.

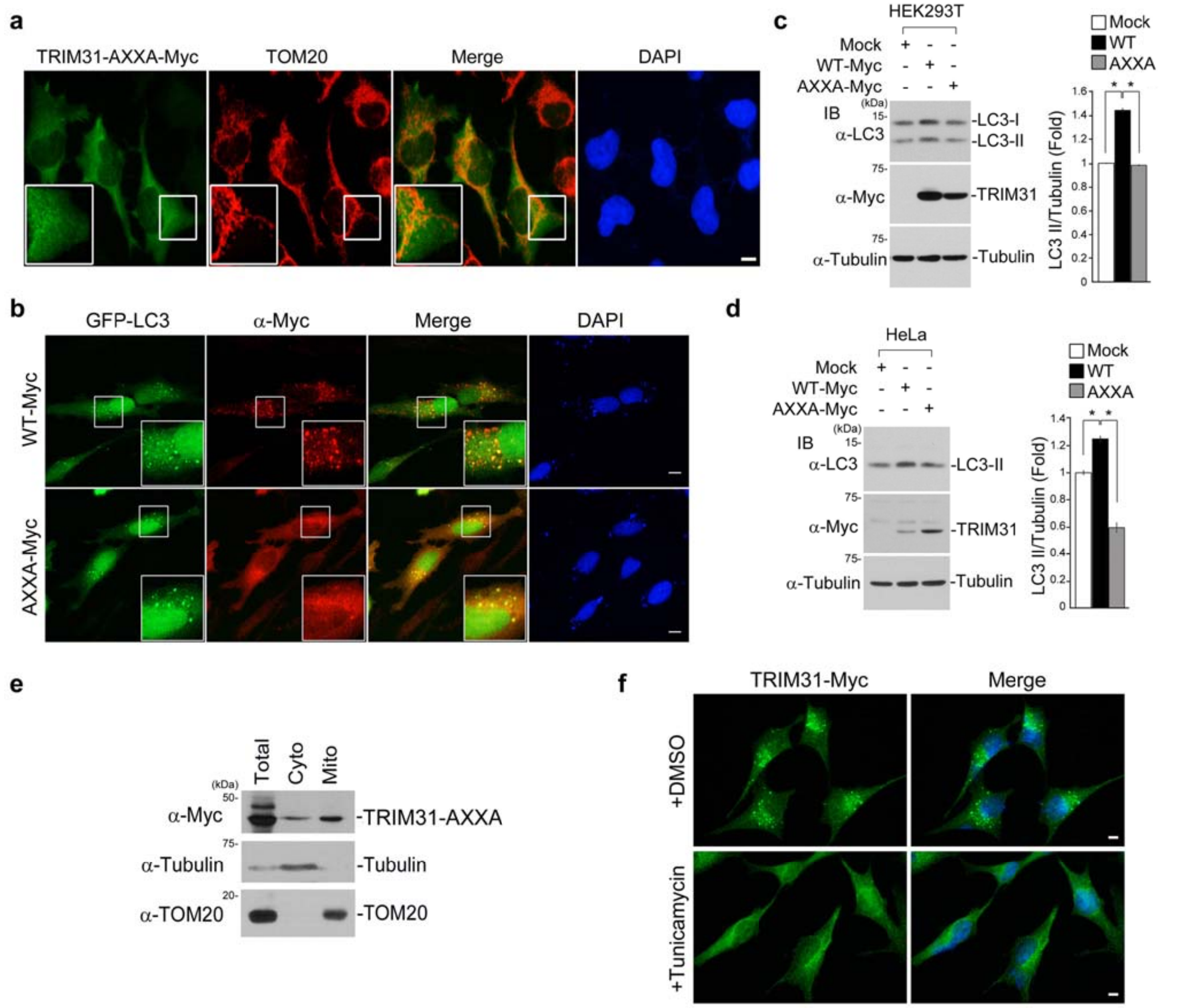

Supplementary Figure 8. Palmitoylation is essential for TRIM31-mediated autophagic activity. (**a**) Mitochondrial localization of TRIM31-AXXA-Myc in HeLa cells stably expressing TRIM31-AXXA-Myc. Cells were immunostained with anti-Myc (green) and anti-TOM20 (red) antibodies, followed by counterstaining with DAPI (blue). TOM20 was used as a mitochondrial marker. Scale bars, 5 μm. All data are representative of at least two independent experiments. **(b)** Immunofluorescence assay of HeLa cells cotransfected with GFP-LC3 and each TRIM31-Myc construct. Blue, DAPI. Scale bars, 5 μm. (**c** and **d)** Immunoblot analysis of LC3-II in protein extracts from HEK293T **(c)** or HeLa cells **(d)** transfected with pLHCX (Mock), WT-TRIM31 (WT-Myc), or AXXA-mutated TRIM31-Myc (AXXA-Myc) vectors. *Right graph*, quantification of LC3-II levels normalized against tubulin. *P<0.01 (Student's t-test). (**e**) Mitochondrial localization of AXXA-mutated TRIM31-Myc (AXXA-Myc). Anti-Tubulin and anti-TOM20 antibodies were used to detect the cytosolic (Cyto) and mitochondrial (Mito) fractions, respectively. **(f)** HeLa cells stably expressing TRIM31-Myc were treated for 1 h with DMSO or tunicamycin (20 μg per ml), a compound which inhibits palmitoylation. Cells were immunostained using anti-Myc antibody and analyzed by immunofluorescence assay. Scale bars, 5 μm. All data are representative of at least three independent experiments (mean ± s.d. in **c** and **d**).

Supplementary Figure 9. Ra et al.

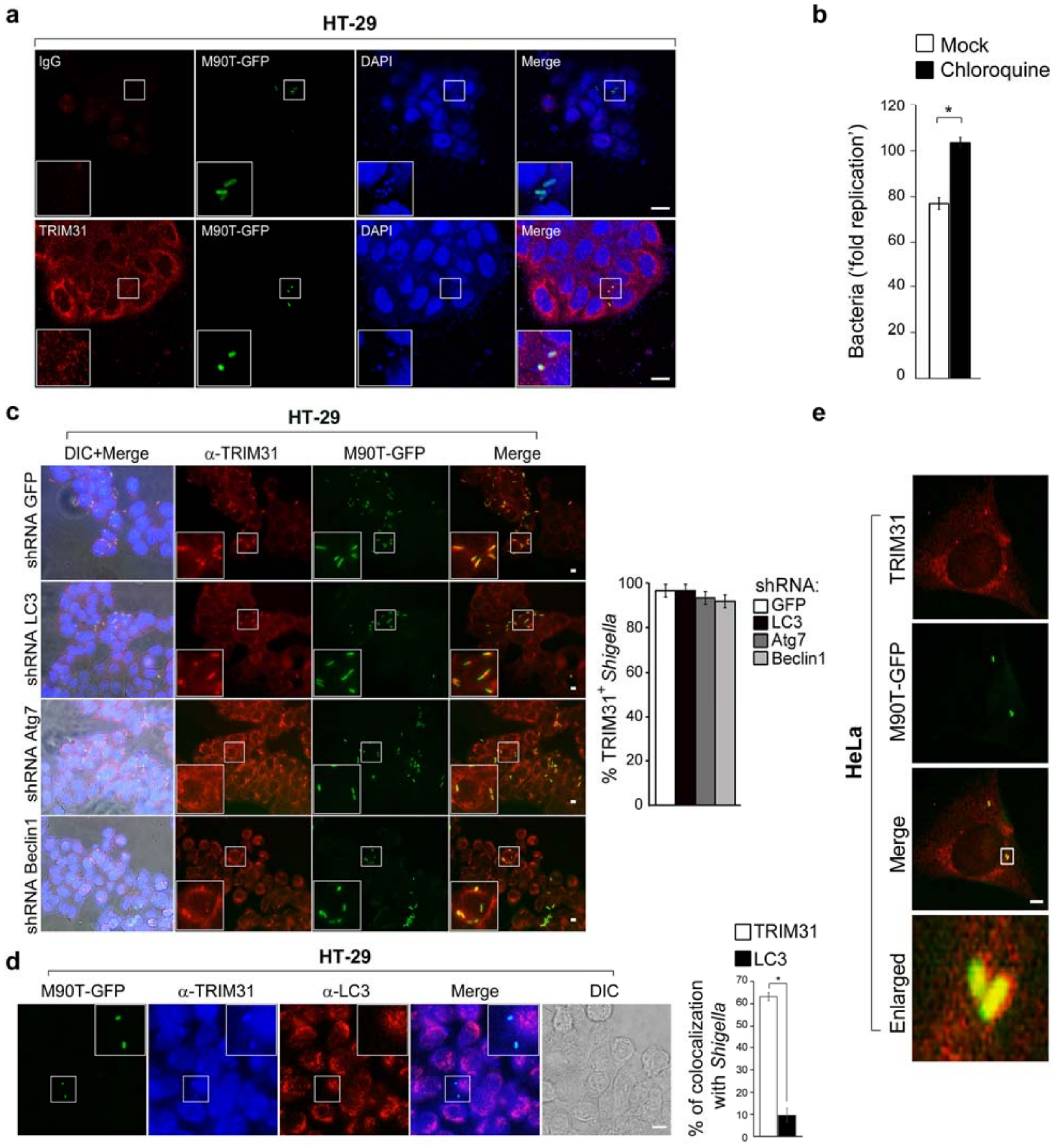

Supplementary Figure 9. Colocalization of endogenous TRIM31 with *Shigella* in HT-29 cells (a) HT-29 cells were infected with GFP-tagged *S. flexneri* M90T (green), washed twice with PBS, and chased in complete media for 1 h with gentamicin (100 µg per ml) to eliminate extracellular bacteria. Cells were fixed and immunostained with anti-TRIM31 antibody (red), followed by counterstaining with DAPI (blue). Scale bar, 5 µm. **(b)** The bacterial recovery assay in HT-29 cells that were left untreated or treated with chloroquine (5 µM) for 4 h. Cells were infected with M90T-GFP and incubated for 2 h with gentamicin (100 µg per ml). *P<0.01 (Student's t-test). **(c)** Immunofluorescence assay of *Shigella*-infected HT-29 cells stably expressing shRNA GFP, shRNA LC3, shRNA Atg7, or shRNA Beclin1. After infection with M90T-GFP (green), cells were incubated with gentamicin (100µg per ml)

for 1 h and immunostained for endogenous TRIM31 (red). DIC, differential interference contrast; Blue, DAPI. Scale bars, 5 μm. *Right Graph*, quantification of TRIM31-positive puncta in HT-29 cells stably expressing shRNA GFP, shRNA LC3, shRNA Atg7, or shRNA Beclin1. **(d)** HT-29 cells infected with M90T-GFP (green) were stained with anti-TRIM31 (blue) and anti-LC3 (red) antibodies. DIC, differential interference contrast. Scale bar, 5 μm. *Right graph*, quantification of TRIM31- or LC3-positive bacteria in HT-29 cells. *P<0.01 (Student's *t*-test). **(e)** TRIM31-overexpressing HeLa cells infected for 1 h with M90T-GFP (green) were stained with anti-TRIM31 antibody (red) and then visualized by IFA. Scale bar, 5 μm. All data are representative of at least two independent experiments (mean ± s.d. in **b,** and **d**).

Supplementary Figure 10. Ra et al.

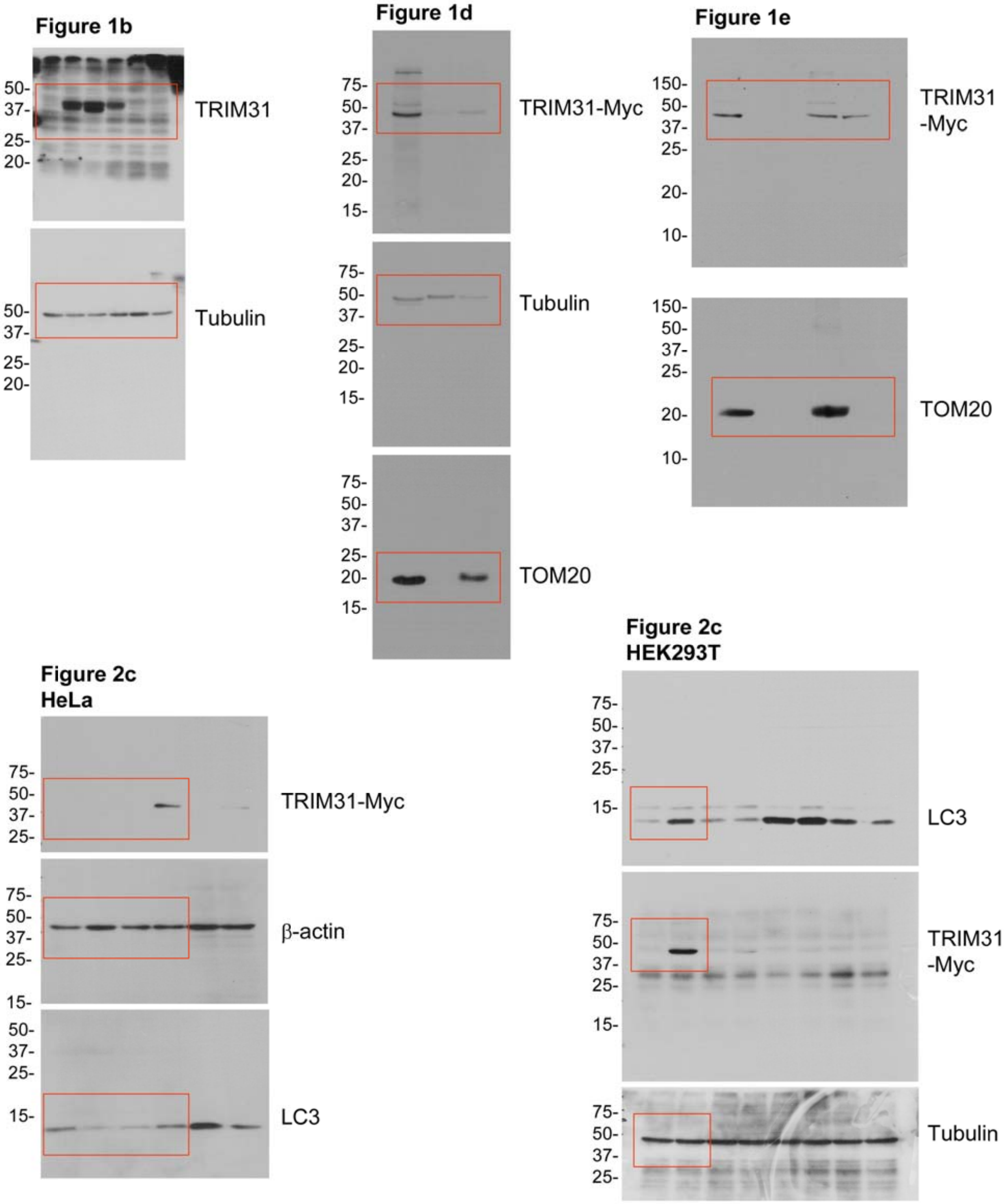

Figure 2e

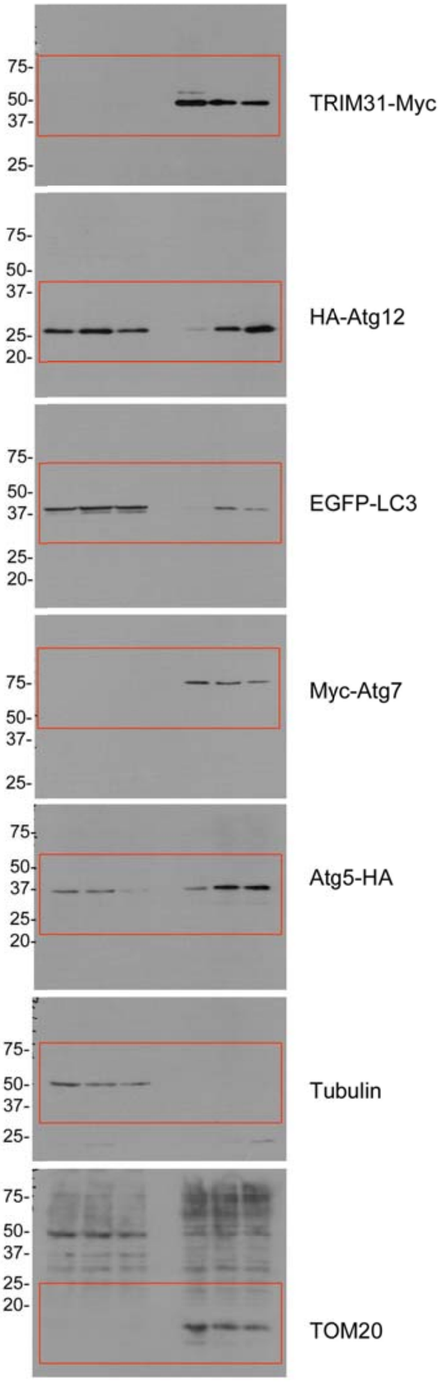

Figure 3f

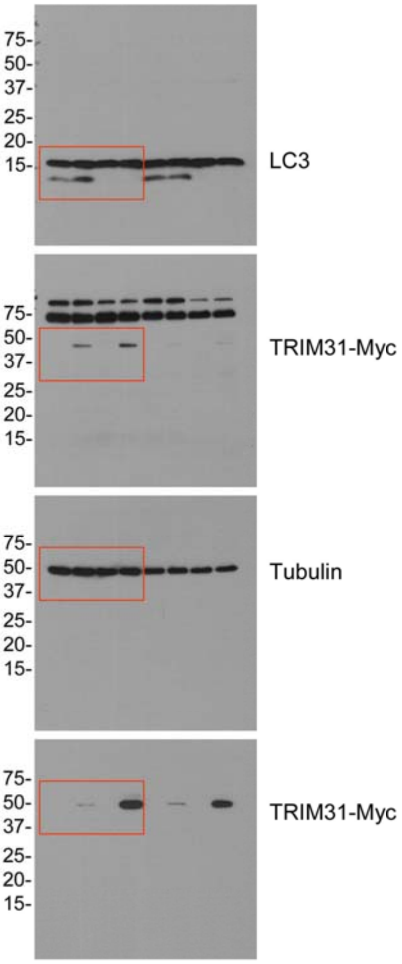

Figure 6b

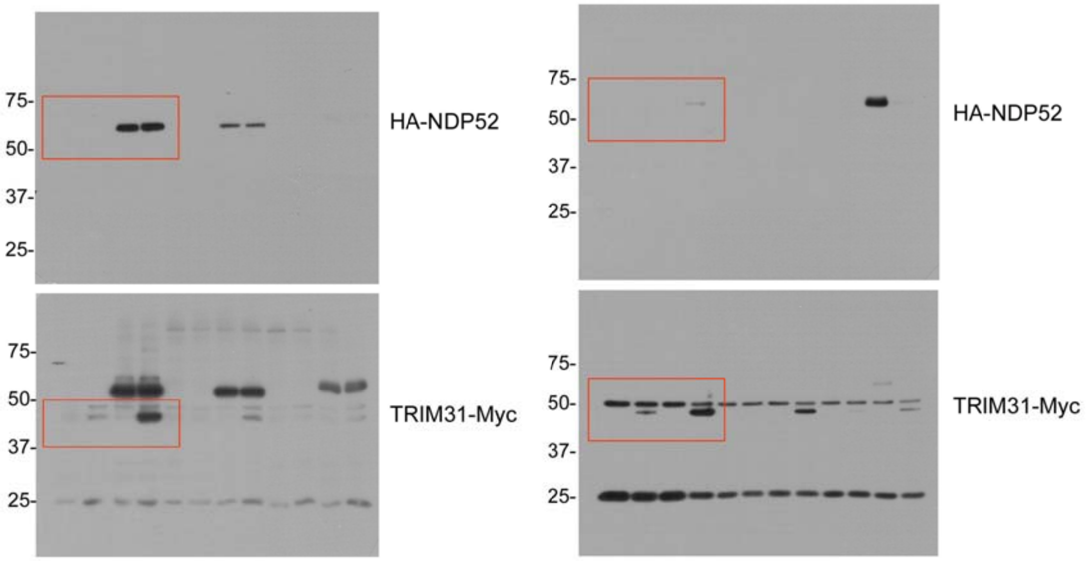

Supplementary Figure 10. List of uncropped images of western blot analysis

Supplementary Table 1. Sequences of oligomers for shRNA, mutagenesis, and RT-PCR

**shRNA**	
shRNA TRIM31 sense	5′-GATCCGAGCAGATCCAAGTCTTGCTTCAAGAGAGCAA GACTTGGATCTGCTCTTTTTTGGAAA-3′
shRNA TRIM31 antisense	5′-AGCTTTTCCAAAAAAGAGCAGATCCAAGTCTTGCTCTC TTGAAGCAAGACTTGGATCTGCTCG-3′
shRNA GFP sense	5′-GATCCGCAAGCTGACCCTGAAGTTCCTC GAGGAACTTCAGGGTCAGCTTGCTTTTTTGGAAA-3′
shRNA GFP antisense	5′-AGCTTTTCCAAAAAAGCAAGCTGACCCTGAAGTTCCTC GAGGAACTTCAGGGTCAGCTTGCGG-3′
shRNA LC3B sense	5′-GATCCGCTGAGATCGATCAGTTCATCTCGAGATGAAC TGATCGATCTCAGTTTTTTGGAAA-3′
shRNA LC3B antisense	5′-AGCTTTTCCAAAAAACTGAGATCGATCAGTTCATCTC GAGATGAACTGATCGATCTCAGCG-3′
shRNA Atg7 sense	5′-GATCCGGAGTCACAGCTCTTCCTTCTCGAGAAGGAAG AGCTGTGACTCCTTTTTTGGAAA-3′
shRNA Atg7 antisense	5′-AGCTTTTCCAAAAAAGGAGTCACAGCTCTTCCTTCTC GAGAAGGAAGAGCTGTGACTCCG-3′
shRNA Beclin1 sense	5′-GATCCGCAGTTTGGCACAATCAATACTCGAGTATTGA TTGTGCCAAACTGTTTTTTGGAAA-3′
shRNA Beclin1 antisense	5′-AGCTTTTCCAAAAAACAGTTTGGCACAATCAATACTCG AGTATTGATTGTGCCAAACTGCG-3′
**TRIM31 mutagenesis**	
TRIM31 sense	5′-GCTAAGCTTGCCACCATGGCCAGTGGGCAGTTTGTGAA-3′
TRIM31 antisense	5′-GCTAGATCTGCTTGAAGGAACCTTACAAAACCAAG-3′
ΔN sense	5′-ATTAAGCTTGCCACCATGAGGAAGAACGCAATC AGGTTCAACT-3′
ΔC antisense	5′-ATTAGATCTGAATTTTTTCAGGCTCCCTGTGATG-3′
ΔRING sense	5′-GCAAGAGGAAGTGATCAGGAAGAACGCAATCAG-3′
ΔRING antisense	5′-CTGATTGCGTTCTTCCTGATCACTTCCTCTTGC-3′
ΔBB sense	5′-CAGTCCAAAAGGAAAGAGGCTACAGAAGAAGCTGCCC AGAATTATCAG-3′
ΔBB antisense	5′-CTGATAATTCTGGGCAGCTTCTTCTGTAGCCTCTTTCC TTTTGGACTG-3′
AXXA sense	5′-ATGGGAAGTTCCTCGCTTTTGTGGCTCGTGA ATCCAAGGA-3′
AXXA antisense	5′-TCCTTGGATTCACGAGCCACAAAAGCGAGG AACTTCCCAT-3′
**RT-PCR**	
TRIM31 sense	5′-CGTAAGGAAGAACGCAATCAGGTT-3′
TRIM31 antisense	5′-CTTCTCATGTTCTACCTGGTCCG-3′

LC3B sense	5′-ATGCCGTCGGAGAAGACCTTCA-3′
LC3B antisense	5′-TTACACTGACAATTTCATCCCGAACGT-3′
Beclin1 sense	5′-TGTCACCATCCAGGAACTC-3′
Beclin1 antisense	5′-CTGTTGGCACTTTCTGTGG-3′
Atg7 sense	5′-GGAGATTCAACCAGAGACC-3′
Atg7 antisense	5′-GCACAAGCCCAAGAGAGG-3′
IE1 sense	5′-CTGATAATCCTGACGAGGGC-3′
IE1 antisense	5′-TGCTCCTTGATTCTATGCCG-3′
GAPDH sense	5′-TGATGACATCAAGAAGGTGGTGAA-3′
GAPDH antisense	5′-TCCTTGGAGGCCATGTGGGCCAT-3′

Supplementary Table 2. Detailed CD patient information

IHC (n=8)

Patient #	Sex	Age	Duration(yr)	Location	Behavior	Treatment	Severity*
1	M	43	9	Ileocolonic	Penetrating	ADA, AZA	Moderate
2	M	43	6	Ileocolonic	Penetrating	-	Moderate
3	M	23	7	Ileocolonic	Penetrating	AZA	Moderate
4	F	33	15	Ileocolonic	Penetrating	-	Moderate
5	M	32	8	Ileocolonic	Stricturing	IFX	Moderate
6	F	35	10	Ileocolonic	Penetrating	AZA	Moderate
7	F	41	16	Ileal	Penetrating	-	Moderate
8	M	49	11	Ileocolonic	Penetrating	ADA	Moderate

*Remission: CDAI score <150. Mild: CDAI score of 150-219. Moderate: CDAI score of 220-450. Severe: CDAI score >450
CDAI, Crohn's Disease Activity Index; ADA, adalimumab; AZA, azathioprine; IFX, infliximab

RT-PCR (n=3)

Patient #	Sex	Age	Location	Behavior	Treatment	Activity*
1	M	17	Ileocolonic	Inflammatory	Remicade	Active phase
2	M	21	Ileocolonic	Inflammatory	-	Active phase
3	M	28	Ileocolonic	Inflammatory	-	Active phase

*Remission phase: CDAI score <150. Active phase: CDAI score≥150
CDAI, Crohn's Disease Activity Index; AZA, azathioprine
